# Application of Nucleic Acid Frameworks in the Construction of Nanostructures and Cascade Biocatalysts: Recent Progress and Perspective

**DOI:** 10.3389/fbioe.2021.792489

**Published:** 2022-01-07

**Authors:** Gan Zhu, Ping Song, Jing Wu, Minglan Luo, Zhipeng Chen, Tingjian Chen

**Affiliations:** MOE International Joint Research Laboratory on Synthetic Biology and Medicines, School of Biology and Biological Engineering, South China University of Technology, Guangzhou, China

**Keywords:** nucleic acids, unnatural base pairs, unnatural nucleic acids, nanostructures, cascade biocatalysts

## Abstract

Nucleic acids underlie the storage and retrieval of genetic information literally in all living organisms, and also provide us excellent materials for making artificial nanostructures and scaffolds for constructing multi-enzyme systems with outstanding performance in catalyzing various cascade reactions, due to their highly diverse and yet controllable structures, which are well determined by their sequences. The introduction of unnatural moieties into nucleic acids dramatically increased the diversity of sequences, structures, and properties of the nucleic acids, which undoubtedly expanded the toolbox for making nanomaterials and scaffolds of multi-enzyme systems. In this article, we first introduce the molecular structures and properties of nucleic acids and their unnatural derivatives. Then we summarized representative artificial nanomaterials made of nucleic acids, as well as their properties, functions, and application. We next review recent progress on constructing multi-enzyme systems with nucleic acid structures as scaffolds for cascade biocatalyst. Finally, we discuss the future direction of applying nucleic acid frameworks in the construction of nanomaterials and multi-enzyme molecular machines, with the potential contribution that unnatural nucleic acids may make to this field highlighted.

## Introduction

Natural nucleic acids, including deoxyribonucleic acid (DNA) and ribonucleic acid (RNA), laid the material foundation for the storage and retrieval of genetic information in all living organisms on this planet. Nucleic acids demonstrated very unique properties and advantages to be used for constructing nanomaterials and scaffolds of multi-enzyme systems. For example, the structures of nucleic acids are well determined by their sequences, which allows straightforward control of their structures by customized sequence design (Seeman, 1990; Lu and Olson, 2003). The feature that single-stranded nucleic acids can bind with their complementary strands with very good affinity and specificity also enables precise assembly of complex structures with nucleic acids (Niemeyer et al., 1999). Meanwhile, chemical modifications of the nucleic acids and proteins that bind specific sequences of nucleic acids provide a number of simple means to immobilize and organize proteins on the nucleic acid scaffold (Verma and Eckstein, 1998; Zheng and Wang, 2011; Patterson et al., 2014; Spicer and Davis, 2014). Furthermore, good biocompatibility of nucleic acids and the fact that nucleic acids can be efficiently produced inside the cells by living organisms make the nucleic acid scaffolds not only enzyme-friendly frameworks with various applications *in vitro*, but also powerful tools for *in vivo* assembly of nanostructures and multi-enzyme molecular machines (Wilner et al., 2009; Simmel, 2012; Schoffelen and van Hest, 2013; Shi et al., 2018a). In recent years, modified or unnatural moieties, including unnatural nucleobases and unnatural sugar/phosphate skeletons, have been designed, synthesized, and introduced into nucleic acids, which significantly expanded the sequence space, properties, functions, and applications of nucleic acids, and some of them have already been employed in the construction of nucleic acid frameworks with novel properties (Ochoa and Milam, 2020). Hopefully, these unnatural moieties will make nucleic acid frameworks more competitive scaffolds for the construction of nanostructures and multi-enzyme systems in the future.

In this review article, we start from a brief introduction of the molecular structures and properties of natural and unnatural nucleic acids. Then the approaches and recent progresses on fabricating and applying complex nanostructures and multi-enzyme systems with nucleic acid scaffolds *in vitro* and *in vivo* are summarized and discussed. A perspective on future development and application of novel nucleic acid frameworks in building nanostructures and multi-enzyme systems is provided in the end, with the potential contribution of unnatural nucleic acids highlighted.

## Nucleic Acids and Their Unnatural Derivatives

### Natural Nucleic Acids

**FIGURE 1:**
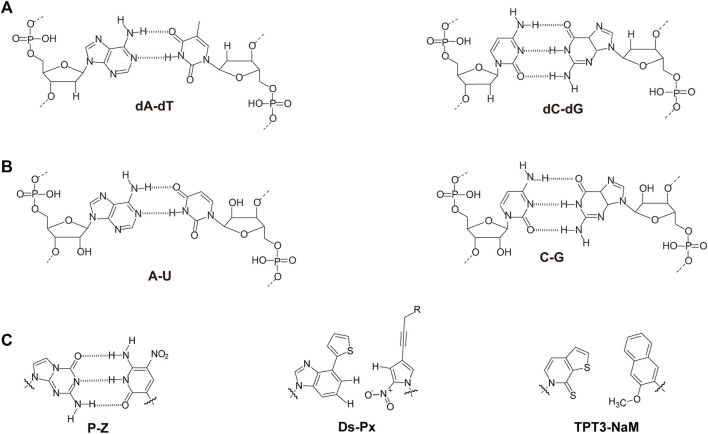
Chemical structures of monomeric units for natural nucleic acids and representative unnatural base pairs. **(A)** Monomeric units of DNA. **(B)** Monomeric units of RNA. **(C)** Representative unnatural base pairs.

Nucleic acids have chain structures formed by the polymerization of nucleotide monomers through 3′-5′ phosphodiester linkages, and two nucleic acid strands with complementary sequences can hybridize with each other to form an anti-parallel duplex with a helical structure under proper conditions. As shown in Figures 1A,B, a typical nucleotide unit consists of a nitrogenous base (nucleobase), a pentose, and a phosphodiester group. The pentose is deoxyribose in DNA, and ribose in RNA, which contributes to the significant differences between DNA and RNA in chemical structures and properties (Narayana and Weiss, 2009; Mooers and Singh, 2011). For example, the 2′-OH group in the ribose of RNA is very easy to get deprotonated and then serves as a good nucleophile under various conditions, which contributes to the catalytic activities of some RNA molecules. The 2′-OH group can also lock a double-stranded RNA into a compact A-like helix, which leads to an increased melting temperature compared with that of a B-form double-stranded DNA (Anosova et al., 2016). In DNA, the nucleobases include adenine (A), thymine (T), guanine (G), and cytosine (C), while in RNA, T is replaced by uracil (U). Watson-Crick base pairing of these nucleobases governs the precise transfer of genetic information during DNA replication and RNA transcription, which makes DNA and RNA not only perfect materials for the storage and retrieval of genetic information, but also excellent structural polymers with amplifiable sequences for the construction of artificial architectures. The highly specific hybridization of a DNA or RNA sequence with its complementary sequence also allows precise assembly of DNA or RNA strands into various microarchitectures, ranging from simple linear structures to fairly sophisticated three-dimensional structures, some of which have been used as scaffolds to build multi-enzyme systems (Fu et al., 2014; Sachdeva et al., 2014; Ngo et al., 2016). Moreover, the development of phosphoramidite chemistry for automated *de novo* synthesis of DNA and the invention of polymerase chain reaction (PCR) technology for exponential amplification of DNA have already laid the technical foundations for large-scale preparation of DNA, which is the prerequisite to extensively use DNA as a structural material (Caruthers, 1985; Saiki et al., 1988).

### Unnatural Base Pairs

Recently, the development of unnatural base pairs (UBPs) has greatly expanded the genetic alphabet (Kimoto and Hirao, 2020). Introduction of UBPs pairing orthogonally to natural base pairs into DNA obviously increased the possible sequences, structures, properties, and functions of DNA, and optimization of them for natural-like efficiencies of replication, transcription and translation further expanded their applications. Figure 1C demonstrates the chemical structures of three most predominant UBPs. The pairing of UBP P-Z, which was developed by Benner group, is based on hydrogen bonding with a different pattern from those of natural base pairs, while the pairing of UBPs TPT3-NaM and Ds-Px, which were developed by Romesberg group and Hirao group respectively, is based on hydrophobic and packing forces (Kimoto et al., 2008; Li et al., 2014). These UBPs have been shown to be very efficient for PCR amplification and *in vitro* transcription, and thus significantly increased the storage capacities of DNA and RNA for amplifiable and retrievable information (Li et al., 2014). Moreover, these UBPs have already been successfully used in various *in vitro* applications, ranging from site-specific labeling of nucleic acids to selection for affinity reagents (Lavergne et al., 2016; Futami et al., 2019; Kimoto et al., 2019; Zhang et al., 2020; Matsunaga et al., 2021; Miao et al., 2021; Niu et al., 2021).

Most remarkably, UBP TPT3-NaM have been used in the construction of the first six-letter semi-synthetic organism, and proven to be very efficient for *in vivo* replication, transcription, and even translation (Malyshev et al., 2014; Zhang et al., 2017a; Fischer et al., 2020). These exciting discoveries immediately enabled countless potential *in vivo* applications of UBPs, including *in vivo* site-specific labeling of nucleic acids to simultaneous introduction of multiple different unnatural amino acids into proteins. More detailed information about development and application of unnatural base pairs has been summarized in other specific reviews (Feldman and Romesberg, 2018; Kimoto and Hirao, 2020).

### Nucleic Acids With Unnatural Sugars

**FIGURE 2:**
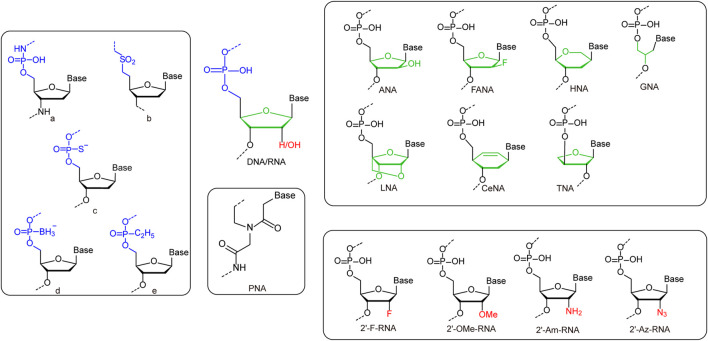
Representative unnatural modifications on sugar and phosphate backbone of nucleic acids. Left rounded rectangle: a: Monomeric unit of N3′-P5′ phosphoramidate-linked nucleotides. b: Monomeric unit of sulfone-linked nucleotides. c: Monomeric unit of phosphorothioate-linked nucleotides. d: Monomeric unit of boranephosphonate-linked nucleotides. e: Monomeric unit of ethylphosphonate diester-linked nucleotides. Upper right rounded rectangle: Monomers of nucleic acids with deoxyribose/ribose replaced by unnatural sugars. Lower right rounded rectangle: Monomers of nucleic acids with 2′-substitutions on deoxyribose/ribose. Middle rounded rectangle: Monomers of PNA.

While the incorporation of UBPs increases the sequence diversity and sequence-related functionality of nucleic acids, the chemical modification or substitution of sugar or phosphate moieties usually leads to dramatic change of overall structures and properties of nucleic acids (Figure 2 and Table 1). 2′-modification is the most explored modification on the sugar of nucleic acids. By chemical synthesis, the hydrogens or hydroxyl groups at 2′-position of deoxyriboses in DNA or riboses in RNA are replaced with various atoms or groups, including fluorine (2′-F), chlorine (2′-Cl), azido (2′-Az), amino (2′-Am), and methoxy (2′-OMe) groups. These modifications usually lead to increased resistance of nucleic acids against nuclease degradation in biological solutions, due to good substrate specificities of natural nucleases (Burmeister et al., 2005; Liu et al., 2017). It was also reported that the 2′-F and 2′-OMe modifications on DNA lead to significant increase of duplex stability and T_m_ value, mainly due to a conformation shift to an RNA:RNA duplex like structure (Table 1). Egli et al. demonstrated that the strongly electronegative and poorly polarizable fluorine atom leads to increased Watson-Crick base pairing strength, and possibly π-π stacking interactions as well, which contributes to the higher pairing affinity and stability of the duplex (Manoharan et al., 2011; Pallan et al., 2011).

**TABLE 1:** Structures and characteristics of backbone-modified unnatural nucleic acids.

Nucleic acid duplex	Structure	Characteristics and properties	References
DNA:DNA	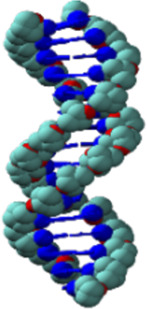 PDB ID: 3BSE	A/B-form right-handed duplex or Z-form left-handed duplex	Narayana and Weiss (2009)
RNA:RNA	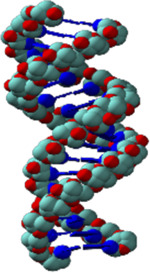 PDB ID: 4S3N	A-form right-handed duplex	Mooers and Singh (2011), Donovan et al. (2015)
		Higher thermal stability than DNA duplex	
ANA:RNA	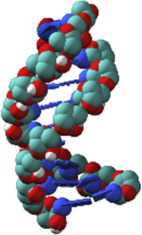 PDB ID: 2KP3	A/B-form right-handed duplex	Watts et al. (2010)
		Less stable than DNA duplex	
FANA:RNA	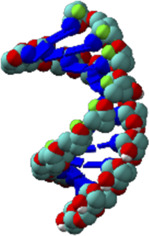 PDB ID: 2KP4	A/B-form right-handed duplex	Martin-Pintado et al. (2012), Anosova et al. (2016)
		Higher thermal stability than ANA duplex	
2′-F-RNA:2′-F-RNA	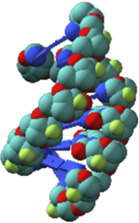 PDB ID: 3P4A	A-form right-handed duplex	Pallan et al. (2011)
		Enhanced thermal stability	
2′-OMe-RNA:2′-OMe-RNA	Not deposited in PDB	Higher resistance to nuclease than DNA or RNA duplex	Burmeister et al. (2005), Eichert et al. (2010), Liu et al. (2017)
		Higher thermal stability than RNA duplex	
LNA:LNA	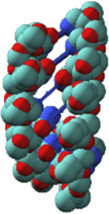 PDB ID: 2X2Q	A-like right-handed duplex	Eichert et al. (2010), Pallan et al. (2011)
		Higher thermal stability than 2′-F-RNA or 2′-OMe-RNA duplex	
GNA:GNA	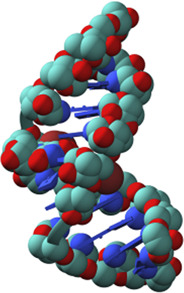 PDB ID: 5V1K	N-type or M-type right-handed duplex	Johnson et al. (2011)
PNA:PNA	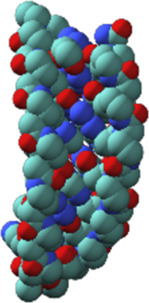 PDB ID: 2K4G	P-type right-handed or left-handed duplex	He et al. (2008)
TNA:TNA	Not deposited in PDB	A-like right-handed duplex	Ebert et al. (2008)
HNA:RNA	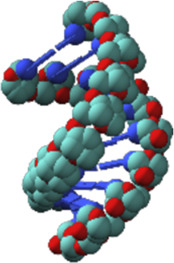 PDB ID: 2BJ6	A-form right-handed duplex	Declercq et al. (2002)
		Higher melting temperature than HNA:DNA duplex	
CeNA:CeNA	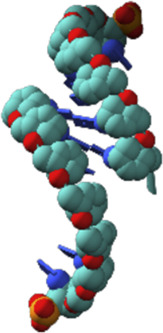 PDB ID: 2H0N	(Mirrored) A-form left-handed duplex	Robeyns et al. (2008)

Other than direct modifications on deoxyribose or ribose, various unnatural sugars were also introduced into the backbones of nucleic acids to replace deoxyribose or ribose, and different unnatural sugars in the backbones usually lead to bigger differences in the structures and properties of nucleic acids (Table 1). Replacement of deoxyribose in DNA with arabinose or its 2′-fluoro derivatives leads to the production of arabinonucleic acids (ANA) or 2′-F arabinonucleic acids (FANA). Despite the close similarity between ANA and FANA, their properties are strikingly different, due to the different groups at the 2′-position. In FANA, the 2′-OH of ANA is replaced by a smaller and more electronegative fluorine atom, which favors the formation of intra-residue C2′-F … H8-C pseudohydrogen bonds (Watts et al., 2010; Martin-Pintado et al., 2012). Consequently, FANA:FANA duplex shows significantly higher thermostability than ANA:ANA duplex, although both of them are B-like duplexes. Locked nucleic acid (LNA) duplex has a right-handed A-like helical structure, and each sugar of LNA contains a 2′-O, 4′-C methylene bridge, which “locks” the sugar in the C3′-endo conformation, and thus endows the duplex with higher stability (Eichert et al., 2010). In glycol nucleic acid (GNA), the sugar is replaced by a three-carbon propylene glycol. GNA can form highly stable antiparallel duplexes with N-type or M-type helical structures (Johnson et al., 2011). The sugar moieties in the backbone of R-(L)-threofuranosyl-(3′-2′) nucleic acid (TNA) are R-(L)-threoses, which are linked through 2′-3′ phosphodiester bridges. Although the introduction of R-(L)-threose into the backbone is a drastic structural modification for nucleic acids, TNA can also form stable homoduplex or heteroduplex with DNA or RNA (Ebert et al., 2008). Hexitol nucleic acid (HNA) has a phosphorylated 1′, 5′-anhydrohexitol backbone, and forms an A-like helical structure. A single-stranded HNA can form duplex with another single-stranded HNA, a single-stranded RNA, or a single-stranded DNA with following order of duplex stability: HNA:HNA > HNA:RNA > HNA:DNA (Declercq et al., 2002). Cyclohexene nucleic acid (CeNA) is a nucleic acid also with a six-membered ring in the backbone, while a rigid double bond in the ring makes its structure less flexible. CeNA can also form antiparallel homoduplex *via* Watson-Crick base pairing, but with a left-handed helix type (Robeyns et al., 2008). Peptide nucleic acid (PNA), which was initially described by Nielsen and coworkers, is a synthetic analogue of DNA that has a pseudopeptide backbone instead of a sugar-phosphate backbone. Different from DNA, which forms A-, B-, or Z-form duplexes, PNA forms duplex with a helical structure named P-type (He et al., 2008). Due to their highly diverse structures and properties, nucleic acids with unnatural sugars have already found a lot of applications in biotechnology or biomedicine, ranging from selection of stable affinity reagents to production of novel catalysts (Taylor et al., 2015; Zhang et al., 2020; Matsunaga et al., 2021; Wang et al., 2021b).

### Nucleic Acids With Unnatural Phosphates

The phosphate group in the backbone of nucleic acids has also been chemically modified or substituted with various anionic, cationic, or electroneutral groups for altered properties (Arangundy-Franklin et al., 2019). For example, a series of modified oligonucleotides were developed by replacing non-bridging oxygen atoms in the phosphate group with other atoms or groups, such as S^−^ and BH_3_^−^, to introduce properties for enhanced biological activities (Eckstein, 2014; Kumar and Caruthers, 2020). Recently, Holliger and coworkers replaced one of the non-bridging oxygen atoms in the phosphate with an alkyl group, which led to the production of P-alkyl phosphonate nucleic acid (phNA) with an uncharged backbone (Arangundy-Franklin et al., 2019). The enzymatic synthesis and evolution of these nucleic acids were also accomplished with engineered polymerases. More thorough changes have also been made to the whole bridges between sugars in the backbone. For example, Gryaznov and co-workers developed oligonucleotide N3′-P5′ phosphoramidate, in which the 3′-oxygen of each nucleoside was replaced with a 3′-nitrogen (Chen et al., 1995). This nucleic acid was found to have great nuclease resistance and superior thermodynamic stability, and thus has great potential for therapeutic and other applications. In another early case, Benner and co-workers synthesized nonionic sulfone-linked RNA analogs, rSNAs, by replacing the phosphodiester moieties of RNA with dimethylene sulfone units, and found that rSNAs have many interesting characteristics (Richert et al., 1996).

### Wild Type or Engineered Polymerases for the Recognition and Synthesis of Unnatural Nucleic Acids

Having efficient polymerases is the prerequisite for enzymatic synthesis, amplification, and even evolution of unnatural nucleic acids for different applications. During the design and synthesis of unnatural base pairs, the polymerase recognition of them has already been taken into consideration very well, so the predominant unnatural base pairs, such as TPT3-NaM, P-Z, Ds-Px, can be efficiently replicated and transcribed by many of the natural DNA and RNA polymerases (Table 2). Since natural polymerases usually have poor recognition and synthesis activities for backbone-modified nucleic acids, a lot of efforts have been made to develop engineered polymerases for efficient synthesis of these unnatural nucleic acids. In these efforts, thermophilic single-subunit DNA polymerases were usually employed as starting point for polymerase engineering. Romesberg and co-workers have developed a modified phage display platform for directed polymerase evolution, and successfully applied it to evolve the Stoffel fragment of Taq DNA polymerase for efficient transcription, reverse transcription, and amplification of DNAs with 2′-substitutions, including 2′-F, 2′-OMe, 2′-Am, 2′-Az, 2′-Cl-NTPs and ANA (Table 2) (Chen et al., 2016; Chen and Romesberg, 2017). In an earlier work, Szostak group reported that Therminator DNA polymerase mutant 9°N (A485L) could efficiently synthesize TNA oligonucleotides using threonucleoside triphosphates as substrates (Ichida et al., 2005). Holliger and co-workers developed a selection strategy called compartmentalized self-tagging (CST) for polymerase evolution, and evolved several variants of TgoT DNA polymerase, which demonstrated outstanding synthesis or reverse transcription activities for different unnatural nucleic acids, including HNA, CeNA, ANA, FANA, TNA, and HNA (Pinheiro et al., 2012). In a later work, they further engineered one of these TgoT mutants (RT521L) to yield mutant PGV2, which allows efficient template-directed synthesis of a 57-nucleotide (nt) phNA oligomer (Arangundy-Franklin et al., 2019). Obika and co-workers developed KOD DNA polymerase mutants, KOD DGLNK and KOD DLK, which are able to efficiently synthesize LNAs from DNA templates, or reverse transcribe LNAs back into DNAs, respectively (Hoshino et al., 2020). Remarkably, KOD DGLNK can also produce full-length 2′-OMe-DNA products from DNA templates with good efficiency.

**TABLE 2:** Summary of predominant polymerases for the recognition and synthesis of unnatural nucleic acids.

Polymerase family	Polymerase	Mutation sites	Unnatural nucleic acid products	References
Family A	Taq and Deep Vent DNAP	—	DNA containing UBP NaM-TPT3	Li et al. (2014)
	Deep Vent DNAP	—	DNA containing UBP Ds-Px	Kimoto et al. (2008)
	KlenTaq DNAP	—	DNA containing UBP P-Z, or Ds-Px	Betz et al. (2017); Ouaray et al. (2020)
	T7 RNAP	—	RNA containing unnatural nucleobase NaM or TPT3	Feldman et al. (2019)
	T7 RNAP mutant	Y639F	RNA containing 2′-F-C, U	Padilla and Sousa (1999)
	T7 RNAP mutant	Y639F/H784A	2′-F, 2′-OMe, and 2′-Am-RNA	Padilla and Sousa (2002)
	T7 RNAP mutant	RGVG: R425C, E593G, Y639V, H784G; M5: S430P, N433T, S633P, F849I and F880Y; M6: P266L, S430P, N433T, S633P, F849I and F880Y	2′-F, 2′-OMe, and 2′-Am-RNA	Meyer et al. (2015)
	SF mutant (SFM4-3)	I614E, E615G, V518A, N583S, D655N, E681K, E742Q, M747R	2′-F, 2′-OMe, 2′-Az, 2′-Cl, 2′-Am-DNA/RNA and ANA	Chen et al. (2016)
	SF mutant (SFM4-6)	I614E, E615G, D655N, L657M, E681K, E742N, M747R	2′-F, 2′-OMe, 2′-Az, 2′-Cl, 2′-Am-DNA/RNA and ANA	Chen et al. (2016)
	SF mutant (SFM4-9)	I614E, E615G, N415Y, V518A, D655N, L657M, E681V, E742N, M747R	2′-F, 2′-OMe, 2′-Az, 2′-Cl, 2′-Am-DNA/RNA and ANA	Chen et al. (2016)
Family B	Tgo mutant (Pol6G12)	TgoT: V589A, E609K, I610M, K659Q, E664Q, Q665P, R668K, D669Q, K671H, K674R, T676R, A681S, L704P, E730G	HNA and CeNA	Pinheiro et al. (2012)
	Tgo mutant (PolD4K)	TgoT: L403P, P657T, E658Q, K659H, Y663H, E664K, D669A, K671N, T676I	FANA and ANA	Pinheiro et al. (2012)
	Tgo mutant (PolC7)	TgoT: E654Q, E658Q, K659Q, V661A, E664Q, Q665P, D669A, K671Q, T676K, R709K	LNA and CeNA	Pinheiro et al. (2012)
	Tgo mutant (Tgo RT521L)	TgoT: E429G, I521L, K726R	TNA, phNA	Pinheiro et al. (2012), Arangundy-Franklin et al. (2019)
	9°N exo^−^ mutant	A485L	TNA	Ichida et al. (2005)
	KOD mutant (DGLNK)	N210D, Y409G, A485L, D614N, E664K	LNA, 2′-OMe-RNA	Hoshino et al. (2020)
	Tgo mutant (PGV2)	Tgo RT521L: D455P, 487G, R606V and R613V	phNA	Arangundy-Franklin et al. (2019)

RNA polymerase from T7 phage is the RNA polymerase most extensively engineered for unnatural activities. Y639F mutant of T7 RNA polymerase was reported to be able to use dNTPs, 2′-Am and 2′-F-NTPs as substrates to synthesize modified RNA (Padilla and Sousa, 2002). It was also found that further introduction of H784A mutation into this mutant resulted in better recognition of bulkier 2′-substituents, such as 2′-OMe and 2′-Az-NTPs. Later in 2015, Ellington and co-workers reported several thermostable mutants of T7 RNA polymerase, RGVG, M5 and M6, for the synthesis of 2′-modified RNA with much higher yields (Meyer et al., 2015). All these approaches of engineering polymerases with outstanding unnatural activities enabled efficient enzymatic production of unnatural nucleic acids, and have laid a solid technical foundation for using unnatural nucleic acid elements in the construction of artificial nanostructures and even potential frameworks of multi-enzyme systems.

## Application of Nucleic Acid Frameworks in the Construction of Nanostructures

Taking advantage of aforementioned unique characteristics and properties of nucleic acids, a variety of highly programmable nanostructures have been constructed with different nucleic acid frameworks. In depth exploration of these nanostructures have guided their extensive application in many fields, including biosensing, biomedicine, and biocatalysis.

### Application of DNA Frameworks in the Construction of Nanostructures

**FIGURE 3:**
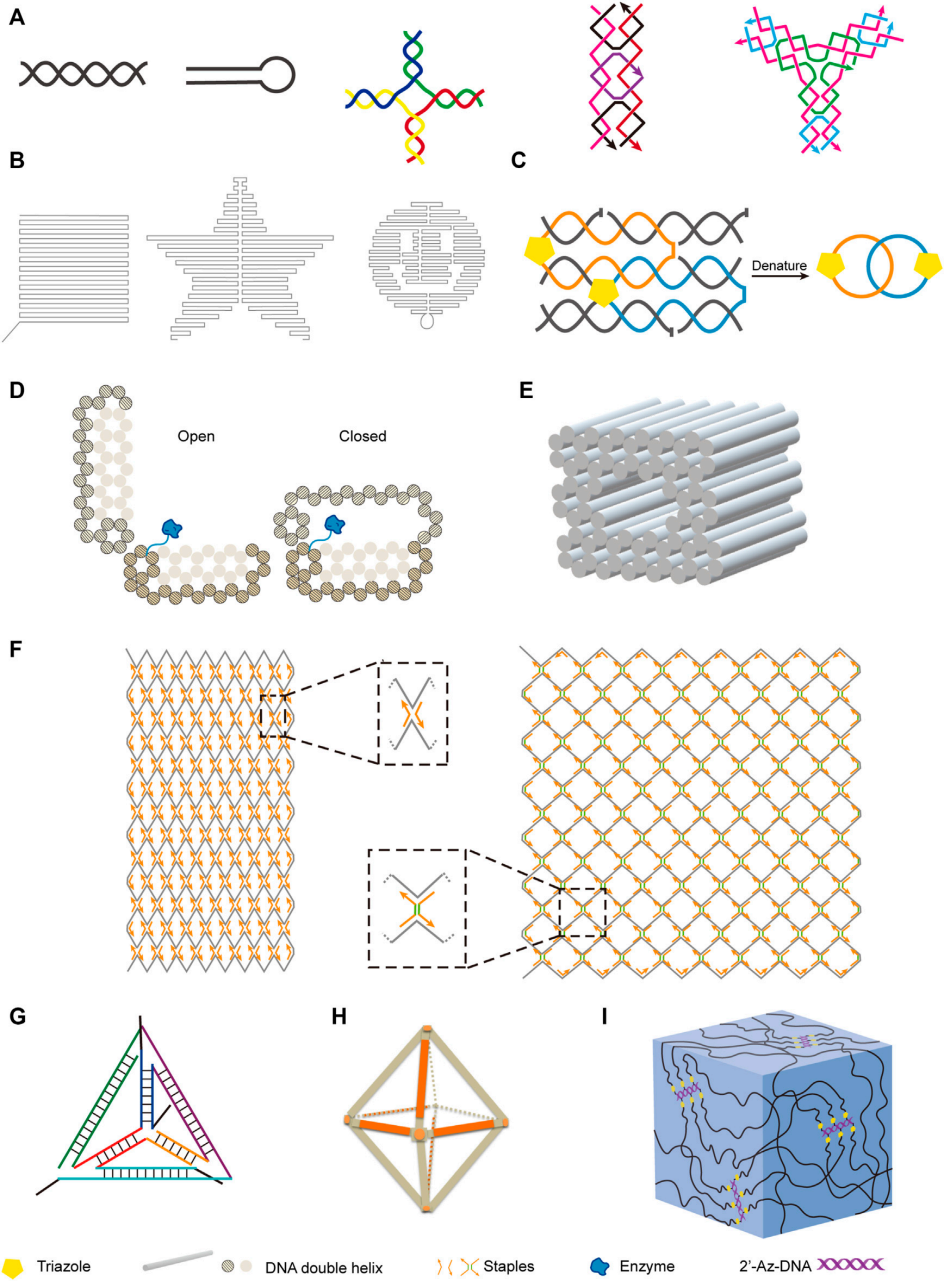
Examples of nanostructures constructed with natural and unnatural nucleic acids. **(A)** Basic units of DNA nanostructures. From left to right: DNA double helix, hairpin, Holliday junction, rectangular DNA tile, Y-shaped DNA tile. **(B)** Two-dimensional DNA origami shapes. From left to right: square, star, smiling face. **(C)** DNA catenane. Four selected neighboring strands of a six-helix bundle were modified with 3′-alkyne and 5′-azide, and intramolecularly cyclized *via* click reaction to form the topologically interlocked structure. **(D)** Enzyme-loaded DNA nanovault in the open and closed states. **(E)** Square nut-shaped DNA origami. **(F)** Rhombic lattice and hexagonal lattice constructed with integration of scaffolded DNA origami and scaffold-free LEGO methods. Representative vertexes are magnified in dotted frames. Staples with or without spacers (shown in green) of different lengths were crucial to modulate the lattice pattern. **(G)** Unnatural nucleic acid tetrahedron. **(H)** FANA octahedron. **(I)** Nucleic acid hydrogel constructed with 2′-azido modified DNA backbone.

DNA is the most explored nucleic acid to be used as material for the construction of artificial structures. The duplex of canonical B-form DNA has a diameter of 2 nm, and the distance between two adjacent base pairs is 0.34 nm, which ideally suits to the construction of nanoscale structures. Taking advantage of precise hybridization between two DNA strands with complementary sequences, DNA can easily form different nano-sized simple structures, including linear double-stranded helices, hairpin structures, kissing complexes, Holliday junctions, and DNA tiles (Figure 3A). In some cases, DNA can also form triplex or quadruplex structures, due to different hydrogen bonding patterns of nucleobases (Rosu et al., 2002; Park et al., 2021). To construct more complicated DNA structures, these simple structural units can be assembled in a defined order, either by hybridizing different DNA strands, or by integrating parts of different structural units into the same strand of DNA. Pioneered by Seeman, various DNA-based nanostructures with higher complexity have been designed and constructed (Seeman, 1982), and these structures retain the inherent characteristics of DNA, including good structural programmability, biocompatibility, and biodegradability. DNA origami technology was then systematically developed, and widely used for bottom-up design and construction of well-defined two-dimensional (2D) and three-dimensional (3D) nanostructures with DNA sequences (Jun et al., 2019a; Jun et al., 2019b).

By assembling DNA motifs in programmed manners on a plain, various 2D nanostructures can be constructed. For example, Rothemund developed a method for making arbitrary 2D shapes with DNA, in which a long single-stranded M13 genomic DNA scaffold was folded by using short DNA oligonucleotides as staple strands to hold it in place (Figure 3B) (Rothemund, 2006). DNA tiles are very useful structural elements in the construction of DNA nanostructures, and have been assembled into 2D lattice or other complex shapes, or closed up to form DNA nanotubes (Sharma et al., 2009; Wei et al., 2012). Introduction of intrastrand or interstrand chemical crosslinking can be helpful to increase the stability of these DNA nanostructures. Cassinelli et al*.* built “chain-armor”-stabilized 6-helix DNA tile tubes with short DNA oligonucleotides, some of which are modified with alkyne and azide on their 3′-ends and 5′-ends respectively (Cassinelli et al., 2015). After assembly of the oligonucleotides, the 3′-ends and 5′-ends of them were covalently connected *via* click reaction (Figure 3C), which led to interlocking of the oligonucleotides and significantly increased stability of the nanostructure under extreme conditions.

Highly programmed assembly of a large number of “DNA bricks”, which are very short DNA oligonucleotides, for the construction of complicated 3D nanostructures has been extensively explored in recent years. Yin group constructed more than a hundred different 3D nanostructures with sophisticated surface features by assembling hundreds of 32-nt ssDNA bricks, each of which can bind to four local neighbors and be independently removed or added (Ke et al., 2012). In some cases, long DNA strands were also employed for the construction of 3D origami structures. Andersen et al*.* constructed a 42 × 36 × 36 nm^3^ cubic box from six DNA sheets, which were fabricated by folding the same circular single-stranded M13 phage genomic DNA with staple DNA strands, and remarkably, the lib of this box can be opened with external key oligonucleotides (Andersen et al., 2009). Gorssi et al*.* designed a DNA nanovault device, which also uses M13 phage genomic DNA as folding scaffold, and this device can be programmed to control enzyme-substrate interactions by inducible conformational changes (Figure 3D) (Schoenit et al., 2021). Shih group applied stapled long ssDNA derived from M13 phage genome to build a series of honeycomb-pleated 3D nanostructures with precisely controlled dimensions and various complex shapes, including monolith, railed bridge, stacked cross, slotted cross, genie bottle and square nut-like shapes (Figure 3E) (Douglas et al., 2009). Shih group also demonstrated that the complex shapes of DNA nanostructures could be twisted or curved by deleting or inserting base pairs at targeted positions, which is very useful in the construction of shapes with curvatures, such as ball-like structures (Dietz et al., 2009; Douglas et al., 2009). Recently, Cui et al. integrated both scaffolded DNA origami method and scaffold-free LEGO method to build 2D and 3D wireframe structures (Cui et al., 2021). By simply changing the DNA staples, they transformed the rhombic lattice made of zigzagged scaffold into larger and more flexible hexagonal lattice with adjustable hexagon units (Figure 3F). They also fabricated hybrid diamond cubic lattices that are able to accommodate AuNPs, and their strategy allows the adjustment of pore volumes according to the size of guest molecules.

While DNA origami nanostructures were constructed with various strategies, Dietz group further assembled them to construct dynamic DNA devices and giant programmable DNA assemblies (Gerling et al., 2015; Wagenbauer et al., 2017). In another work, they demonstrated rapid production of 3D DNA nanostructures by folding the DNA strands at constant temperatures, and the yields could be as high as 100% (Sobczak et al., 2012). They also designed a strategy for mass production of DNA origami components, in which the precursor DNA of the origami components and interspaced Zn^2+^-dependent DNAzyme sequences were incorporated into M13 phage genome, and amplified during the phage propagation (Praetorius et al., 2017). After extraction of the phage genomic DNA, the DNA origami components were self-excised off by the DNAzymes. All these efforts would directly contribute to the more robust and easier construction of versatile DNA nanostructures with lower costs, and enable their large-scale applications in various fields.

Due to their unique properties, DNA nanostructures have great potential to be broadly applied in biotechnology and biomedicine. For example, some DNA nanostructures can penetrate cell membranes without transfection agents for intracellular detection or cargo transport, which provides alternative methods for molecular diagnosis and drug delivery (Jiang et al., 2021). Fan group demonstrated that DNA tetrahedron modified with unmethylated CpG motifs can noninvasively enter macrophage-like cells without transfection agents and induce the secretion of cytokines including tumor necrosis factor-α, interleukin-6 and interleukin-12 (Li et al., 2011). Li et al. designed a DNA origami-based nanorobot, which could precisely respond to the tumor associated molecular trigger, and release drug payload in a controllable manner (Li S. et al., 2018). In this work, a DNA origami sheet was constructed with M13 genomic DNA scaffold and DNA staples, and loaded with thrombin, which could activate coagulation cascade. The DNA origami sheet was then wrapped up and locked with DNA fasteners to form a DNA nanorobot. The fasteners integrated nucleolin aptamer sequences, and could be triggered to open the DNA origami sheet and expose the encapsulated thrombin upon the binding of nucleolin aptamer with nucleolin, which is specifically expressed on the surface of tumor-associated endothelial cells. This DNA nanorobot proved very safe and effective in inhibiting tumor growth in model animals, presumably due to good biocompatibility and precise drug delivery. Krishnan and co-workers constructed artificial myosin filaments using DNA nanotubes as scaffolds, which enabled precise spatial organization of myosin motors and allowed actin filaments to glide along the scaffolds (Hariadi et al., 2015). Based on fluorescence resonance energy transfer (FRET) and pH-sensitive conformation change of designed DNA nanomachines, Krishnan group developed several DNA nanodevices to map pH changes in the cells. In 2009, they reported a DNA nanomachine, I-switch, which could be used to map spatial and temporal pH changes inside living cells (Modi et al., 2009). I-switch consisted of three DNA oligonucleotides, two of which adjacently hybridized to the third one, leaving one nucleotide gap between them and two cytosine-rich overhangs modified with fluorophores unhybridized. Low pH led to the formation of an i-motif between the two overhangs, and resulted in the shift of the I-switch conformation from open state to closed state, which subsequently allowed FRET between the two fluorophores. This I-switch was shown to be very efficient for mapping the spatiotemporal pH changes related to endosome maturation. In another work, they successfully used this I-switch to map the pH changes involved in endocytosis in *Caenorhabditis elegans* (Surana et al., 2011). Later, they simultaneously mapped the pH change in different organelles within the same cell by introducing targeting moieties to direct the DNA nanomachines to enter cells *via* different pathways (Modi et al., 2013). Besides DNA nanodevices that were able to map the pH changes in cells, Krishnan group also developed DNA based fluorescent probes for mapping enzymatic activities with subcellular resolution. These probes contained three functional modules, including an analytes sensing dye, an internal reference dye for ratiometric quantitation, and a targeting moiety to localize the reporter to specific organelle, such as a DNA duplex, a DNA aptamer, or a cholesterol group. Hybridization of complementary DNA strands allowed the stoichiometric assembly of these three functionalities onto the same DNA scaffold. By using distinct sensing dyes that respond to different molecules and specific targeting moieties, they have successfully mapped organellar disulfide exchange, the activity of nitric oxide synthase 3 (NOS3) at the plasma membrane or the trans-Golgi network, and the activity of nitric oxide synthase 2 (NOS2) in phagosomes and endosomes (Dan et al., 2019; Jani et al., 2020; Veetil et al., 2020). Ion concentrations and membrane potential could also be mapped with similar DNA probes incorporating different functional groups and dyes. In 2019, they reported a DNA scaffold-based reporter that is sensitive to both pH and Ca^2+^ concentration, CalipHluor, for the measurement of Ca^2+^ concentrations in specific organelles of the endolysosomal pathway (Narayanaswamy et al., 2019). Recently, they reported a DNA scaffold-based voltmeter that can report the membrane potential of organelles, Voltair (Saminathan et al., 2021). DNA walkers, which can locomote along programed tracks through the processes including DNA strand hybridization, enzymatic cleavage of DNA strands, and DNA strand displacement, are among the most studied dynamic DNA nanodevices, and have been broadly used in various practical applications, such as biosensing, in recent years (Valero and Skugor, 2020; Cha et al., 2015). Dong et al. developed a highly sensitive electrochemical method for the detection of tumor exosomes (Dong et al., 2018). The binding of the aptamers immobilized on the magnetic beads and the exosomes led to the release of three kinds of messenger DNAs, which then hybridized with the DNA probes immobilized on a gold electrode, allowing subsequent exo III digestion of the DNA probes and a decrease in electrochemical signal. Once a probe DNA was digested, the messenger DNA would be released, and hybridize with next adjacent probe DNA. This process went on until no probe DNA left. In this system, the signal was amplified first by the release of multiple messenger DNAs and then by Exo III-assisted probe DNA digestion and messenger DNA recycling, which led to much lower detection limit compared with other methods frequently used. Zhao et al. developed a similar but more comprehensive method for the detection of MCF-7 cell-secreted exosome, in which the employed DNA walker is propelled by DNAzyme-mediated DNA cleavage (Zhao et al., 2019). CD63 aptamers and DNA substrates for a DNAzyme were co-immobilized on magnetic beads, and an EpCAM aptamer linked with the DNAzyme through a swing arm was also added into the system. Simultaneous binding of the CD63 aptamer and the EpCAM aptamer with the target exosome initiated the cleavage of the DNA substrates, releasing product DNA strands that could trigger Exo III-mediated cleavage of probe DNAs immobilized on gold electrodes and an electrochemical signal change similar as previous example. After cleaving a DNA substrate, the DNAzyme went on to bind and cleavage another DNA substrate, which repeated until all the adjacent DNA substrates were cleaved. In another example, Feng et al. developed a sensitive electrochemiluminescence method for the detection of tumor exosomes (Feng et al., 2020). In this method, anchor DNAs containing a nicking recognition sequence and a swing arm partially hybridizing with a CD63 aptamer were co-assembled on a RuSi NP modified electrode. In the presence of exosomes, binding of the aptamer and the exosome led to release of the swing arm and allowed its hybridization with one of the anchor DNAs, which led to the formation of a dsDNA cleavage site for an endonuclease. After the cleavage of an anchor DNA by the endonuclease, the liberated swing arm would continuously locomote to another anchor DNA. The ssDNA products remaining on the electrode then hybridized with complementary ssDNA stands modified with GOx, which catalyzed the oxidation of H_2_O_2_ and eventually led to the decrease of electrochemiluminescence signal. Dynamic platforms or systems combining DNA origami and other components have also found broad practical application recently. For example, Sun et al. developed a DNA origami raft-based platform for real-time imaging of the protein trajectories on 2D fluidic surfaces (Sun et al., 2017). Cholesterol modified double-stranded DNA and DNA origami raft were used for tethering fluorophore-labeled enzymes on the supported lipid bilayer, and both dynamic and static tethering could be achieved with different tethering modes. By using a total internal reflection fluorescence microscope (TIRFM), motions of the enzymes could be monitored in real time, which enabled the imaging of dynamic interactions of proteins. Summary specifically about DNA nanodevices and their applications could be found elsewhere (Simmel, 2007; Krishnan and Simmel, 2011; Kim et al., 2018; Jahanban-Esfahlan et al., 2019; DeLuca et al., 2020; Li et al., 2021a).

More extensive assembly or crosslinking of DNA nanostructures has led to the production of various macroscopic DNA hydrogels. For example, in 2006, Luo group constructed several biocompatible and biodegradable DNA hydrogels by assembling branched DNA monomers, including X, Y, or T-shaped DNA, which was simply done by DNA hybridization and T4 ligation under physiological conditions (Um et al., 2006). Later, they expanded this work by using a restriction enzyme-digested plasmid as a crosslinker to crosslink the X-shaped DNA, which led to the construction of a hydrogel for highly efficient cell-free expression of proteins from the crosslinker plasmid (Park et al., 2009). Much more functional DNA materials, including DNA hydrogels, have been constructed with branched DNA in recent years. For more information specifically about functional DNA materials made from branched DNA and their application in diagnostics, protein engineering, drug and gene delivery, therapeutics, and cell engineering, please refer to a review from Yang and co-workers (Dong et al., 2020). In another approach, Luo group successfully constructed a DNA hydrogel metamaterial with unusual mechanical properties using ϕ29 DNA polymerase-mediated DNA rolling circle amplification (RCA) products (Lee et al., 2012). This work pioneered a series of efforts of constructing functional DNA materials or devices with RCA products. For example, Yang group synthesized a super-soft and super-elastic DNA robot based on magnetic DNA hydrogel fabricated with RCA products (Tang et al., 2020). In this DNA robot, short ssDNA modified magnetic nanoparticles (MNPs) provided permanent crosslinking points for ultra-long ssDNAs obtained from RCA reaction. The hybridization between the short ssDNAs and the ultra-long ssDNAs triggered the secondary amplification and the entanglement among the ultra-long ssDNAs, which led to a dynamic crosslinking. This DNA robot was able to navigate continuously under magnetic force, and was successfully used to deliver cells in a confined space. For more information specifically about DNA materials and devices made from RCA products and their applications, please refer to reviews by Ali et al. (2014), Xu et al. (2021), Yang et al. (2014). Other than branched DNA or RCA products, other simple DNA components can also be used to assemble functional DNA hydrogels. For example, Guo et al. reported the construction of a micro-sized organelle-like hydrogel, which can regulate cellular behaviors, by assembling PCR products produced with chemically crosslinked primers harboring cytosine-rich sequences, which could then bind with each other *via* the formation of i-motifs (Guo et al., 2020). The hydrogel could form *in vivo* in lysosomal acidic microenvironment, which induced the formation of the i-motifs and the transformation of DNA nanoparticles to organelle-like hydrogel*.* The regulation of this organelle-like hydrogel on cellular behaviors was also demonstrated. Recently, the same group reported a DNA network assembled from ssDNA components for capturing T lymphocytes with high purity and viability (Yao et al., 2021). The DNA network integrated three kinds of functional moieties, including aptamers of programmed death-1 (PD-1), CpG oligonucleotides, and complementary sequences, which are responsible for capturing T-cells, enhancing immunotherapy, and enabling the formation of DNA network and inflammatory environment responsive release of T cells and CpG oligonucleotides, respectively. More information specifically about functional DNA hydrogels and their applications can be found elsewhere (Li et al., 2019; Gacanin et al., 2020; Mo et al., 2021).

*In vivo* production of DNA nanostructures may also lead to numerous applications, including scaffold construction for *in vivo* organization of enzymes. However, all the complex DNA nanostructures or materials summarized above were constructed by folding and assembling ssDNA components *in vitro*, while the examples for direct *in vivo* construction of DNA nanostructures are very rare, since *in vivo* production of ssDNA components is not easy and straightforward. In 2008, Yan group reported the *in vivo* amplification and assembly of a simple ssDNA nanostructure, immobile Holliday junction or paranemic cross-over DNA, by integrating its sequence into a phagemid, producing the ssDNA of the phagemid *via* helper phage-mediated rolling circle replication, and crosslinking the self-folded nanostructure with psoralen (Lin et al., 2008). This work indicated that DNA nanotechnology could be well adapted to applications in living cells (Paukstelis and Ellington, 2008)*.* Later, Voigt and coworkers designed a strategy for producing genetic encoded ssDNA and assembling them into DNA nanostructures in living bacteria cells, in which each oligo gene was first transcribed into an RNA containing a 3′-hairpin (HIV Terminator-Binding Site, HTBS), and the ssDNA was then reverse transcribed from the RNA by HIV reverse transcriptase, which was recruited by HTBS (Elbaz et al., 2016). After the RNA regions were degraded by RNases, the ssDNA products self-assembled into nanostructures. In another work of them, a long ssDNA was produced with the same strategy, and then self-cleaved by DNAzymes harbored in its sequence to produce ssDNA components for the assembly of DNA nanoscaffolds in the cells (Alon et al., 2020).

In another effort toward the goal of producing DNA nanoscaffolds *in vivo*, Dietz and coworkers demonstrated that dsDNA could be folded into different nanoscale shapes with the assistance of double transcription activator like (TAL) effector staple proteins (Praetorius and Dietz, 2017). Since both dsDNAs and proteins can be easily and massively produced in living cells, this strategy immediately enabled the *in vivo* construction and application of DNA-protein hybrid nanoscaffolds.

### Application of RNA Frameworks in the Construction of Nanostructures

Rapid development of nucleic acid nanotechnology enabled the design and construction of not only DNA nanostructures, but also RNA nanostructures. Although RNA is less stable than DNA in physiological conditions, it still has unique advantages to be used as a nanostructure material. Different from DNA, single-stranded RNAs can be easily produced by direct transcription from DNA templates *in vivo*, and then spontaneously fold into functional structures, which is very useful for *in vivo* construction, assembly, and application of nucleic acid nanostructures (Li M. et al., 2018). Same as DNA origami technology, RNA origami technology has also been developed to fold and assemble RNA strands into complicated nanostructures (Guo, 2010; Stewart et al., 2017).

Similar as DNA, RNA is able to form various typical secondary and tertiary structural motifs, including three-way junction (3WJ), four-way junction (4WJ), kink-turn motif, hairpins, pseudoknot, C-loops, right-angle motifs, tetraloop-receptors, paranemic motifs, and kissing loops, which can be directly employed in the design and construction of RNA nanostructures (Haque et al., 2018). Early efforts focused on constructing RNA nanostructures with short RNA oligonucleotides. For example, Afonin et al. designed *in silico* and assembled RNA cubic nanostructures with several short RNA oligonucleotides, and found these RNA nanostructures can self-assemble very well in isothermal conditions at 37°C, which immediately suggested a lot of potential applications in biomedicine (Afonin et al., 2010). Mao and co-workers constructed triangular prism and tetragonal prism RNA nanocages using engineered packaging RNAs (pRNAs) of ϕ29 bacteriophage, in which stick ends were added to enable the self-assembly (Hao et al., 2014). Hermann and co-workers fabricated RNA triangles from short oligonucleotides with the guidance of crystal structure (Boerneke et al., 2016). Since the structural motifs of the RNA triangles were designed based on ligand-responsive RNA switches, the assembly process could be controlled by the ligand. Controllable release of the payloads, including functional RNA molecules integrated as structural components, from RNA nanostructures is crucial for practical applications, especially delivery applications, of the RNA nanostructures. Dicer, which is an endoribonuclease crucial in RNA interference process, is responsible for processing double-stranded RNA (dsRNA) precursors to generate functional small RNAs (Park et al., 2011). With in-depth understanding of Dicer processing mechanism, researchers have broadly employed it for the controllable release of functional short RNAs from RNA nanostructure. For example, Ito group designed and constructed branched RNA nanostructures with three- or four-way junction for RNA interference, and Dicer was recruited to transform the assemblies into siRNAs (Nakashima et al., 2011). In another example, Lee group enzymatically synthesized a Y-shaped RNA nanostructure containing Dicer substrates through isothermal rolling circle transcription (RCT) of a circular DNA template, and also successfully demonstrated its application for programmable silencing of multiple genes (Jang et al., 2018).

Construction of RNA origami nanostructures with longer and less RNA strands is attractive, since it will make the transcription process simpler and the *in vivo* construction and application of nanostructures more feasible. Geary et al*.* designed RNA tiles fabricated with a single-stranded RNA, and these RNA tiles can self-assemble into hexagonal lattices (Geary et al., 2014). Remarkably, these RNA nanostructures can form by a co-transcriptional folding manner. Recently, they developed a tool called RNA Origami Automated Design (ROAD) for constructing RNA origami with expanded structural and functional diversity (Geary et al., 2021). Simmel and co-workers developed a similar tile-based RNA nanostructure extending in three dimensions, which could not only again assemble into a hexagonal plane *via* interaction between kissing loops, but also incorporate out-of-plane functionalization (Chopra et al., 2019). By incorporating an RNA motif containing a 90° bend, which allows perpendicularly positioning of other RNA modules, including aptamers, this work provided potential strategies to spatially organize proteins and even construct artificial multienzyme complexes. In 2017, Han et al*.* reported a unimolecular strategy that could be used to design both single-stranded DNA origami and single-stranded RNA origami, and they successfully constructed an RNA origami with a single-stranded RNA as long as ∼6,000 nts with this strategy (Han et al., 2017). Later, Andersen group constructed a unique octahedron RNA nanodevice embedded with siRNA precursors by stapling short strands to the target mRNA scaffold (Hoiberg et al., 2019). The intrinsic recognition sites for Dicer were incorporated into the structure, and facilitated the release of multiple functional siRNAs. This strategy could be extended to produce different target RNA sequences while embedded with different precursors. The design also provided multiple potential sites for the coupling of targeting agents in the structure, which is helpful to improve the specificity of RNA delivery. Torelli et al. also reported an one-pot strategy for the *in vitro* construction of RNA origami, in which the RNA scaffolds and staples are co-transcriptionally folded into nanoribbons containing split broccoli aptamers that can be lit up upon binding with specific dye at 37°C (Torelli et al., 2020). Sugiyamaand coworkers designed and constructed 7-helix bundled planar (7HB-tile) and 6-helix bundled tubular (6HB-tube) RNA origami structures using a 720 nucleotides single-stranded RNA as scaffold (Endo et al., 2014). Short staple RNA strands were used for folding the RNA nanostructures, and chemical modifications were introduced into the nanostructures by applying nucleobase-modified ribonucleoside triphosphates during transcription of the RNA scaffold. Including sugar ring modifications into the scaffolds of RNA nanostructures is helpful to increase the stability of RNA nanostructures under physiological conditions. For example, LaBean and co-workers reported semi-modified RNA origami structures with increased nuclease resistance and stability during storage, as well as enhanced anticoagulant activity (Krissanaprasit et al., 2019). The RNA scaffolds for the origami structures was modified by incorporating 2′-fluoro-modified cytosine and uridine during transcription with a T7 RNA polymerase mutant (Y639F). The 2-helix RNA origami framework was designed to provide four tethering sites for aptamers, and origami nanostructures with multiple thrombin aptamers attached to different sites were constructed and characterized. Compared with free aptamers, the RNA origami nanostructures showed significantly higher anticoagulant activity. Later, they successfully expanded this work to the construction of 3- and 4-helix RNA origami nanostructures with six and eight potential tethering sites to display more RNA aptamers (Krissanaprasit et al., 2021).

Similar as the case of DNA, extensive assembly of RNA structural units have led to the production of more macroscopical RNA materials with various practical applications, including functional RNA hydrogels. For example, Zhang and coworkers constructed an RNA-triple-helix hydrogel incorporating CXCR4 siRNA duplexes, the scaffold of which was produced by rolling circle transcription (RCT), for the treatment of triple negative breast cancers (TNBCs) (Ding et al., 2020). Alternatively, hydrogels with functional RNAs may also be constructed by combining traditional polymer hydrogel materials and RNA components, including RNA nanoparticles. For example, Shi et al. built a thermosensitive PLGA-PEG-PLGA hydrogel harboring RNA polygon nanoparticles, which was designed for potential ocular drug delivery, and demonstrated that the employment of the hydrogel significantly increased the retention of the RNA nanoparticles in the eye (Shi et al., 2018b). Rinaldi and co-workers loaded different programmable RNA nanostructures, including rings and cubes, functionalized with dicer substrates onto polyethylenimine coated magnetic nanoparticles, and demonstrated that RNA nanostructures were more efficient for transfection than RNA duplexes, and the magnetic nanoparticles were efficient for protecting and delivering the functional RNA nanostructures into cells (Cruz-Acuna et al., 2018). Yourston et al. demonstrated the application of programmable RNA nanorings for regulating the formation and properties of silver nanoclusters (AgNCs), which was achieved by chelating AgNCs on cytosine-rich DNA fragments embedded in RNA rings (Yourston et al., 2020). The applications of other nucleic acid nanostructures for guiding the production and assembly of inorganic nanomaterials have also been extensively reported in recent years (Liu et al., 2018; Jing et al., 2019; Shang et al., 2020; Wang et al., 2021a). For more information specifically about this topic, please refer to Heuer-Jungemann and Linko (Heuer-Jungemann and Linko, 2021).

Efforts have also been made to fabricate RNA and DNA structural units together to produce novel RNA-DNA hybrid materials that possess characteristics, properties, and functions of both RNA and DNA. For example, Hermann and co-workers reported a self-assembled RNA-DNA hybrid polygonal nanostructure, the formation and stabilization of which were highly dependent on the binding of the DNA aptamer integrated in the nanostructure with its ligand AMP (Chen and Hermann, 2020). Afonin group fabricated RNA-DNA hybrid nanostructures, which could simultaneously release DS RNAs for siRNA generation and DNA decoy of NF-ĸB, for controllable activation of RNAi and regulation of NF-ĸB transcription in human cells (Ke et al., 2019). Remarkably, by changing the orientation of DNA-DNA interaction parts, the shape of the hybrid nanostructures could be easily changed from fiber to triangle with different physiochemical and immunological properties. Lee et al. constructed an ultrasoft DNA-RNA hybrid hydrogel with the products of rolling circle amplification (RCA) and rolling circle transcription (RCT) for the delivery of siRNA-aptamer complex (SAC) (Han et al., 2020). The sequence responsible for the production of the DNA aptamer targeting nucleolin was included in the template of RCA, while the sequence responsible for the production of the siRNA was included in the template of RCT. The fabrication of this hydrogel was achieved by hybridization of the complementary spacer sequences in the RCA product and the RCT product. This novel hydrogel demonstrated good efficiencies for targeting nucleolin overexpressing tumor cells and controllably releasing SAC to regulate gene expression.

While progresses in efficient construction of RNA nanostructures enabled their broad applications in biotechnology and biomedicine, *in vivo* production of RNA nanostructures *via* co-transcriptional assembly has also been demonstrated by several groups, which suggested the potential use of RNA as a scaffold material for organizing functional molecules in living cells. For example, Delebecque et al. assembled programmed RNA scaffolds *in vivo*, and applied them to guide the spatial organization of proteins (Delebecque et al., 2011). More information about RNA nanotechnology can be found in some other more specific reviews (Grabow and Jaeger, 2014; Ohno et al., 2019).

### Application of Unnatural Nucleic Acid Frameworks in the Construction of Nanostructures

In recent years, along with the fast progress in the design and chemical synthesis of unnatural nucleic acids, as well as polymerase engineering for the enzymatic synthesis and amplification of these molecules, construction of nanostructures with novel structures, properties, and functionalities using unnatural nucleic acids becomes feasible. As mentioned above, some unnatural sugar components introduce not only altered structure into nucleic acids, but also significantly increased helix stability. In 2016, Holliger and co-workers reported the construction of tetrahedron structures entirely composed of FANA, 2′-F-RNA, HNA, or CeNA, and also an octahedron structure composed of FANA (Figures 3G,H) (Taylor et al., 2016). As an example for characterizing the properties of these nanostructures, they examined the degradation of HNA tetrahedron in serum-containing cell culture, and the results clearly demonstrated the superior stability of this nanostructure in biological solutions. Recently, Wang et al*.* constructed FANA double crossover nanostructures, and demonstrated that these nanostructures had increased stability and resistance to nuclease degradation and acidic environment, and thus could serve as ideal carriers for the delivery of small molecules into the cells (Wang Q. et al., 2020). Li et al. reported the construction of dodecahedron cages mimicking the genome of Pariacoto virus with both RNA and 2′-F-modified RNA, and these structures also demonstrated great potential to be employed as nanocarriers (Li et al., 2021b). Chandrasekaran et al*.* introduced 2′-5′ linkages into DNA/RNA hybrid nanostructures, and found this modification led to improved resistance to nuclease degradation (Chandrasekaran et al., 2020).

Other than altered structures and properties, the introduction of unnatural components may also lead to an expansion of the functionality for the nucleic acids, and brand new nucleic acid architectures. For example, we constructed bottlebrush like structures by attaching alkyne-modified single-stranded DNA to 2′-Az modified DNA backbones, and applied it for the construction of nanoparticle arrays and a novel nucleic acid hydrogel, which proved to be a good carrier for proteins (Figure 3I) (Chen and Romesberg, 2017).

## Multi-Enzyme Systems Constructed *in Vitro* Based on Nucleic Acid Frameworks and Their Applications

In living organisms, biochemical processes consist of various enzymatic reactions, including countless cascade reactions, which are often catalyzed by multi-enzyme systems. A multi-enzyme system is composed of a series of enzymes that are well organized in a certain spatial order, which facilitates the transfer of reaction intermediates, and thus significantly enhances the overall catalytic activities (Hwang and Lee, 2019). Inspired by the outstanding catalytic performance of natural multi-enzyme systems, numerous artificial multi-enzyme molecular machines have been designed and applied in a variety of scenarios, ranging from bio-catalysis to biopharmaceutical applications (Zhang et al., 2018; Bergquist et al., 2020). In these artificial multi-enzyme systems, the scaffolds that were used to immobilize and organize the enzymes were prepared from many different materials, including inorganic materials, organic molecules, proteins, and nucleic acids (Jia et al., 2014; Xu K. et al., 2020).

One of the most explored applications of artificial structures composed of nucleic acids is building scaffolds to direct the organization of molecules, including proteins. The outstanding programmability, high diversity, and good biocompatibility of nucleic acid structures make them perfect scaffold materials for elaborate construction of multi-enzyme systems with abundant strategies. While the mature technologies for solid-phase or enzymatic synthesis of oligonucleotides and efficient amplification of genetically encoded DNA or RNA in living cells enable rapid production of massive nucleic acid strands with various lengths, the increasing abundance of methods for precise assembly of these strands and coupling them to proteins makes the spatial organization of enzymes with the guidance of nucleic acid scaffolds manageable and attractive.

### Multi-Enzyme Systems Constructed Based on Simple DNA Scaffolds

**FIGURE 4:**
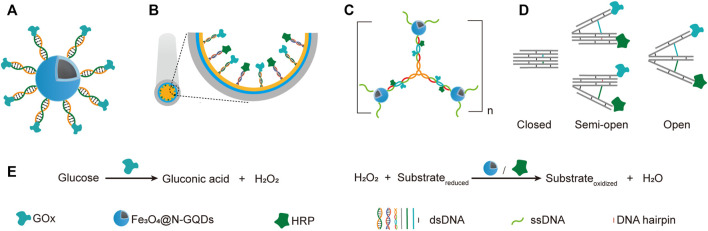
Application of simple DNA scaffolds in the fabrication of designable systems for cascade catalysis. **(A)** DNA directed multi-catalyst system. Ferriferous oxide nanocomposites functionalized with nitrogen-doped graphene quantum dots (Fe_3_O_4_@N-GQDs) served as peroxidases. **(B)** Capillary electrophoresis (CE)-integrated immobilized enzyme reactor (IMER). NH_2_-ssDNA was used to modify the capillary and subsequently direct the anchor of complementary ssDNA-coupled GOX and HRP. **(C)** DNA nanocompartments with encapsulated GOx/HRP dual-enzyme system. **(D)** Switchable trident-shaped DNA nanoactuator with GOx/HRP system, the states of which can be switched by DNA strand displacement reaction. **(E)** The schemes of reactions catalyzed by GOx and HRP or Fe_3_O_4_@N-GQDs.

The simplest way of applying DNA scaffolds for multi-enzyme system construction is to use an ssDNA template-containing scaffold to guide the immobilization and organization of multiple enzymes that are coupled to short DNA oligonucleotides, which are complementary to, and can hybrid specifically with the ssDNA templates in the same scaffold (Niemeyer et al., 1999). Various chemical reactions have been developed for efficient production of DNA-protein conjugates, which promoted the broad application of this strategy. For example, through DNA-directed immobilization, Yang group successfully fabricated a novel multi-catalyst system with glucose oxidase (GOx) immobilized on ferriferous oxide nanocomposites functionalized with nitrogen-doped graphene quantum dots (Fe_3_O_4_@N-GQD magnetic NPs), which mimicked peroxidase (Shen et al., 2019a). GOx was conjugated with a 5′-thiol-modified single-stranded DNA, which is complementary to another single-stranded DNA immobilized on Fe_3_O_4_@N-GQD magnetic NPs through glutaraldehyde. The nanozyme-enzyme system was then obtained through the hybridization of the two complementary ssDNAs (Figure 4A,E).

Later, they described another strategy for the construction of nanozyme-enzyme system, in which the GOx was first immobilized onto magnetic nanoparticles with similar DNA scaffold, and the product nanoparticles were then encapsulated with a layer of nucleotide coordinated polymer (NCP), which was composed of AMP and Ce^3+^ (Shen et al., 2019b). The NCP shield not only prevented the denaturation and detachment of GOx, but also served as a peroxidase. In another work, they demonstrated the DNA-directed assembly of a multi-enzyme system with highly controllable enzyme ratio, in which the regulation of enzyme ratio was achieved by adjusting the ratio of functional groups used for coupling different guide DNA strands on the nanoparticles (Yang et al., 2017). Other than spherical nanoparticles, surfaces of other shapes may also be used for immobilizing DNA-guided multi-enzyme systems to adapt different application scenarios. For example, Li et al. built a capillary electrophoresis-integrated immobilized enzyme reactor (CE-IMER), in which the ssDNA strands for directing the immobilization of complementary ssDNA-coupled GOx and HRP were fixed on the inner surface of a capillary (Figure 4B,E) (Li et al., 2020).

In addition to linear DNA strands, Y-shaped DNA structure was also used as scaffold to construct multi-enzyme system. For example, Song et al. constructed a GOx/HRP multi-enzyme biocatalyst using a Y-shaped DNA scaffold (Song et al., 2019). In this system, The GOx and HRP proteins were immobilized onto the ends of two arms of the Y-shaped DNA scaffold, and the complex was then encapsulated into zeolite imidazolate framework-8. Y-shaped DNA can also be used as a crosslinker to build giant structures, including those with immobilized multi-enzyme systems on them. A DNA nanocompartment with encapsulated GOx/HRP dual-enzyme system was constructed by crosslinking Y-shaped DNA scaffolds with ssDNA-coated magnetic nanoparticles (Figure 4C,E) (Song J. et al., 2020). Each arm of the Y-shaped DNA scaffolds contains a pair of immobilized GOx and HRP, as well as an ssDNA tail in the end, which is complementary to the ssDNA immobilized on the magnetic nanoparticles. Remarkably, the *k*_*cat*_ of immobilized enzymes was found to be 16.6-folds higher than that of free enzymes.

Multi-enzyme systems with dynamic behaviors are attractive due to their capability of regulating catalytic performance in response to environmental changes (Sweetlove and Fernie, 2018). Programmed hybridization of DNA strands not only enables the construction of diverse nanostructures, but also is increasingly applied for the construction of dynamic devices mainly by introducing DNA strand-displacement reactions (Zhang and Seelig, 2011). Xing et al. reported a self-assembled dynamic and reconfigurable trident-shaped DNA (TS DNA) nanoactuator, in which enzymes were tethered to the arms of the trident DNA scaffold (Figure 4D,E) (Xing et al., 2018). The cascade catalytic efficiency of the dual-enzyme system could be regulated by the switch of opened, semi-opened, and closed states of the TS DNA nanoactuator, which corresponded to different distances between the enzymes. Reversible allosteric motion of the arms was achieved by adding the “thermodynamic driver” DNA strands, which led to a shift between stem-loop structure and linear duplex of the DNA spacers between the arms *via* strand displacement reaction.

**FIGURE 5:**
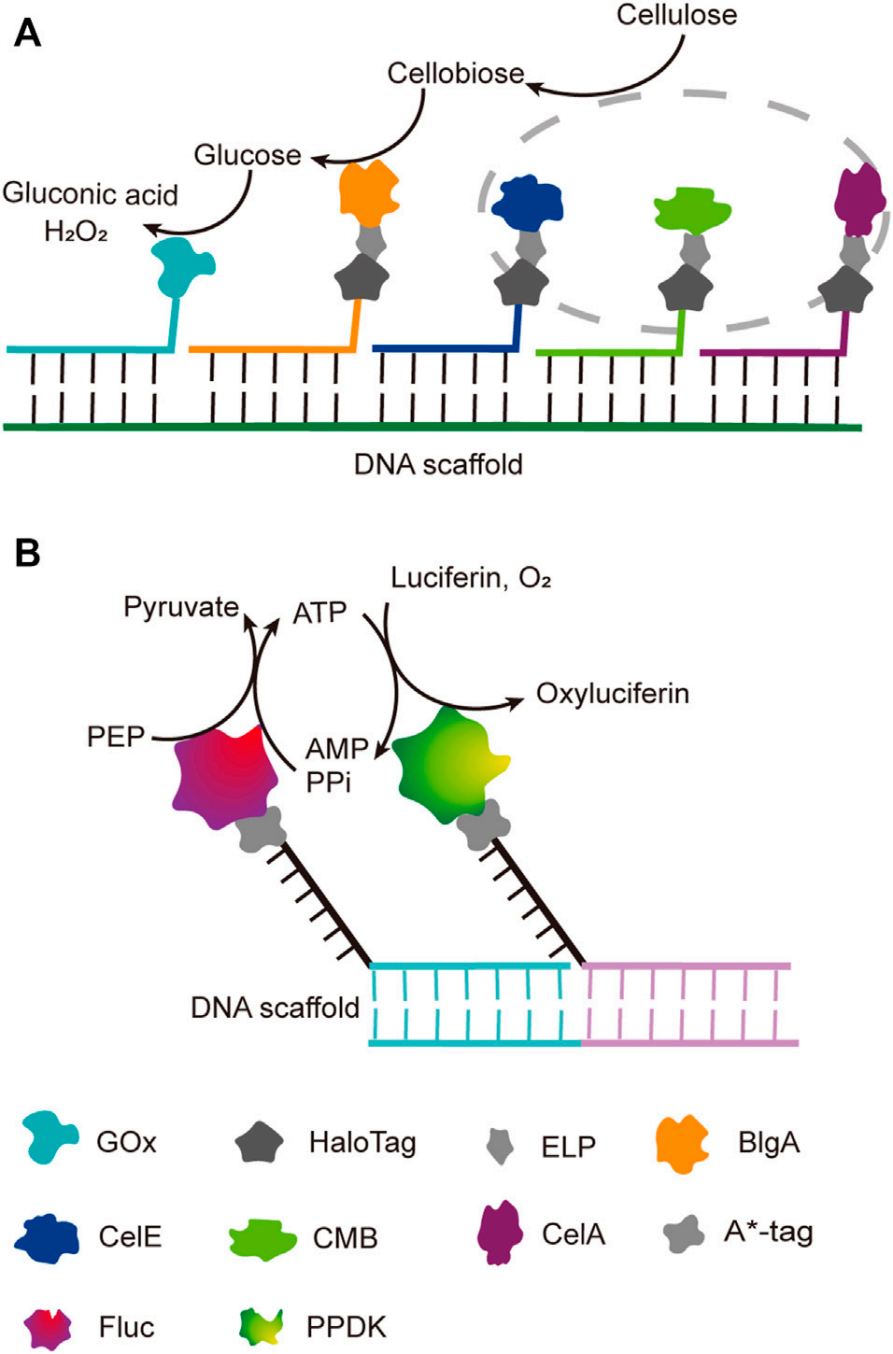
DNA-guided assembly of multi-enzyme systems *via* hybridization of complementary strands. **(A)** Five-enzyme cascade for the conversion of cellulose to gluconic acid. Three cellulosomal components (in grey dot circle), CelA, CelE and CBM, were employed to convert cellulose to disaccharide first; ELP tag was incorporated between each cellulosomal component and HaloTag for simple purification. **(B)** Dual-enzyme system of luciferase (Fluc) and pyruvate orthophosphate dikinase (PPDK). A*-tag cleaves the replication origin sequence of UX174 phage DNA (A* recognition sequence) and covalently binds to the 5′-end of the cleaved cleavage site. ELP: Elastin like polypeptide, CelE: Exoglucanase, CelA: Endoglucanase, CBM: Carbohydrate binding module, BglA: β-glucosidase, FLuc: Luciferase, PPDK: Pyruvate orthophosphate dikinase.

Benefiting from the good orthogonality and high affinity, the orthogonal binding of protein tags and their ligands has been successfully applied in one pot assembly of multiple enzyme-tag fusion proteins on ligand-modified DNA scaffolds. Chen et al. reported a five-component enzyme fuel cell constructed with the guidance of a DNA scaffold for direct conversion of cellulose to gluconic acid and H_2_O_2_ (Chen et al., 2017). All protein components, except for GOx, were genetically fused to a HaloTag, which was then coupled with a chlorohexane group attached to the ssDNA strand for later site-specific hybridization with an ssDNA template (Figure 5A). With similar HaloTag-mediated DNA hybridization strategy, they also reported the construction of an artificial cellulosome by assembling four protein components on a long ssDNA template produced by rolling circle amplification, which led to 2-fold improvement of cellulose hydrolysis (Sun and Chen, 2016).

### Multi-Enzyme Systems Constructed Based on the Binding of DNA Scaffolds and DNA Binding Proteins

**FIGURE 6:**
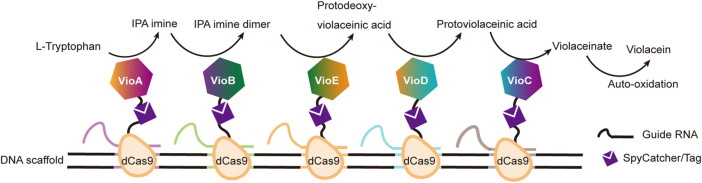
Construction of efficient violacein biosynthesis pathway with five-enzyme complexes based on DNA scaffolds and dCas9 proteins, which bind with specific dsDNA sequences under the guidance of different guide RNAs. VioA: Flavin-dependent L-tryptophan oxidase; VioB: 2-Imino-3-(indol-3-yl) propanoate dimerase; VioC: Violacein synthase; VioD: Protodeoxyviolaceinate monooxygenase; VioE: Violacein biosynthetic enzyme.

DNA binding proteins recognize and bind to specific sequences on single-stranded or double-stranded DNAs (Zheng and Wang, 2011). The fusion of an enzyme to a DNA-binding protein allows convenient immobilization of this enzyme onto a DNA scaffold without any chemical modification, and a multi-enzyme system can be simply constructed with a DNA scaffold harboring multiple recognition sequences of DNA binding proteins. For example, Mashimo et al. co-localized firefly luciferase (Fluc) and pyruvate orthophosphate dikinase (PPDK), each of which was genetically fused with an ssDNA binding protein, A*-tag, on a DNA scaffold constructed through strand hybridization. Enhanced light emission resulted from cascade bioluminescent reaction suggested that the efficiency of the reaction was improved by assembling Fluc and PPDK on the same DNA scaffold (Figure 5B) (Mashimo et al., 2018). Proteins or protein-RNA complexes that can bind with specific dsDNA sequences, including transcription activator-like effectors (TALEs), zinc finger proteins, Cas9/gRNA complexes, have been broadly used in targeted genome editing or gene expression regulation, and also provided powerful tools for the programmable organization of enzymes on a DNA scaffold (Wiedenheft et al., 2012; Qiu et al., 2018). Clustered regularly interspaced short palindromic repeat (CRISPR)/Cas systems are adaptive immune systems of archaea and bacteria species, and have been extensively explored, engineered, and applied for precise genome editing or nucleic acid detection in recent years (Adli, 2018). Cas9 protein is a key component of Type II CRISPR systems, and contains two nuclease domains, which are respectively responsible for the cleavage of target DNA strand and displaced non-complementary DNA strand upon the guide RNA-directed binding of target dsDNA sequence (Wiedenheft et al., 2012). dCas9 is a Cas9 variant in which the dsDNA cleavage activity is ablated *via* mutations at the catalytic sites, while the ability of binding target dsDNA sequence remains (Brocken et al., 2018). Berkman et al. employed dCas9/gRNAs to guide the sequence-specific binding of a SpyTag and a SnoopTag onto a dsDNA scaffold, and a SpyCatcher and a SnoopCatcher, which were fused to two cellulosome components, CBD and CelA respectively, then orthogonally coupled with these two Tags, resulting in co-immobilization of CBD and CelA on the DNA scaffold (Berckman and Chen, 2020). By using this dual-component system, the reducing sugar production was increased for 2.8-fold compared with that of unassembled enzymes. Similarly, Lim et al. demonstrated a five component multi-enzyme complex for the biosynthesis of violacein, in which SpyCatcher/Tag-dCas9/gRNAs guided the immobilization of the components at assigned sites on the DNA scaffold (Figure 6) (Lim et al., 2020). They also demonstrated that the violacein production varied significantly with the proximity of the enzymes. Besides dsDNA, DNA origami nanostructures with protruding DNA-binding protein recognition sequences were also used as scaffolds to construct multi-enzyme complex, and an example will be introduced in the end of next section.

### Multi-Enzyme Systems Constructed Based on Complex DNA Nanostructures

**FIGURE 7:**
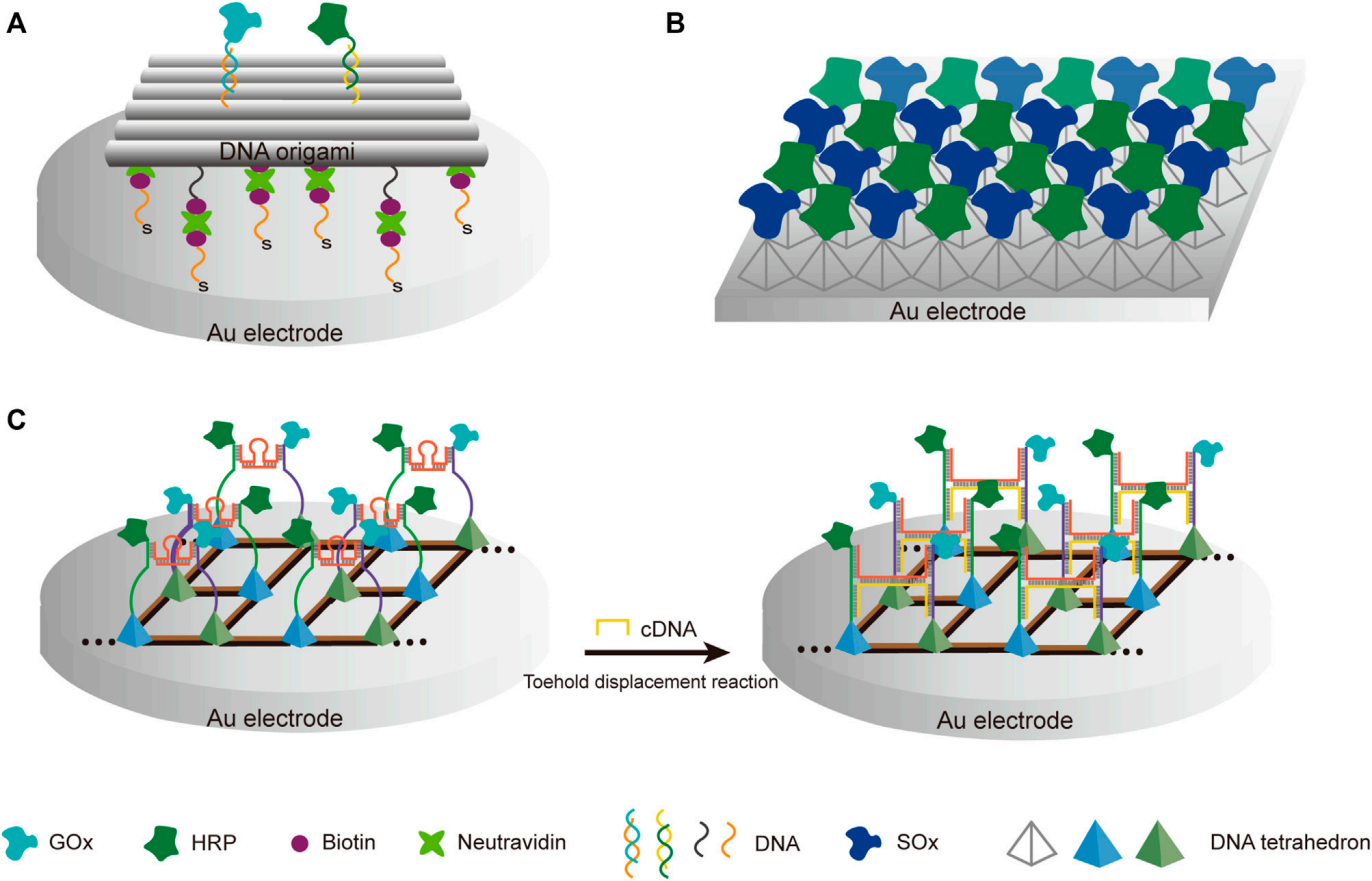
Construction of electrochemical biosensors with DNA scaffold-based multi-enzyme systems. **(A)** Biohybrid device with GOx and HRP assembled on gold electrode through thiol-modified DNA origami scaffold. **(B)** Bulk enzyme heterojunction (BEH)-based sarcosine sensor. Sox and HRP enzymes were immobilized and distributed on a layer of DNA tetrahedrons. **(C)** GOx/HRP dual-enzyme system fabricated with lattice-like framework made of interconnected DNA tetrahedrons as electrochemical biosensor. cDNA converted from target cycling amplification initiated the toehold displacement reaction, and led to the switch of the cascade system from semi-optimal arrangement to optimal arrangement with a 10 nm distance between GOx and HRP.

While DNA scaffolds with simple structures have proven useful in the construction of multi-enzyme systems, higher complexity and programmability of complex DNA nanostructures can benefit the building of more sophisticated and controllable multi-enzyme machineries. Fan group created a biomimetic device, in which a thiol-modified rectangular DNA origami was used to guide the assembly of GOx/HRP enzymatic cascade on gold electrodes (Figure 7A) (Ge et al., 2019). In this device, the enzymatic reaction was translated into a current signal that could be directly recorded, and the use of a carefully designed DNA scaffold allowed programmable adjustment of the precise distance between the enzymes. An irreplaceable advantage of using complex DNA nanostructures or their assemblies to construct multi-enzyme systems is that the enzymes can be easily organized in a highly programmable 2D or even 3D array. Song et al. reported a sarcosine sensor based on bulk enzyme heterojunction (BEH), in which thiolated tetrahedral DNA nanostructures were used to build a uniform layer of SOx/HRP dual-enzyme systems on the surface of electrochemical biosensors (Figure 7B) (Song P. et al., 2020). The good controllability of the enzyme spatial distribution was verified by scanning electron microscope, using gold nanoparticles as substitutes of the enzymes. Wang et al. assembled a lattice-like framework with interconnected DNA tetrahedrons on the surface of an electrode, and immobilized GOx or HRP-coupled DNA strands, which were bridged with tunable DNA strands, on top vertices of these tetrahedrons (Wang et al., 2020a). Sensitive thrombin detection was achieved by finely tuning the arrangement of GOx and HRP with cDNA strands, which were complementary to the bridge DNA strands, and could be released from bead-immobilized thrombin aptamers in the presence of thrombin (Figure 7C). DNA hydrogels can also provide ideal frameworks for the immobilization and organization of multiple enzymes for cascade catalysis. For example, Xiang et al. reported the construction of a DNA hydrogel *via* enzymatic polymerization of DNA building blocks harboring poly-A and poly-T tails and the application of this hydrogel on fabricating a tri-enzyme system for lactose/glucose detection (Xiang et al., 2016).

**FIGURE 8:**
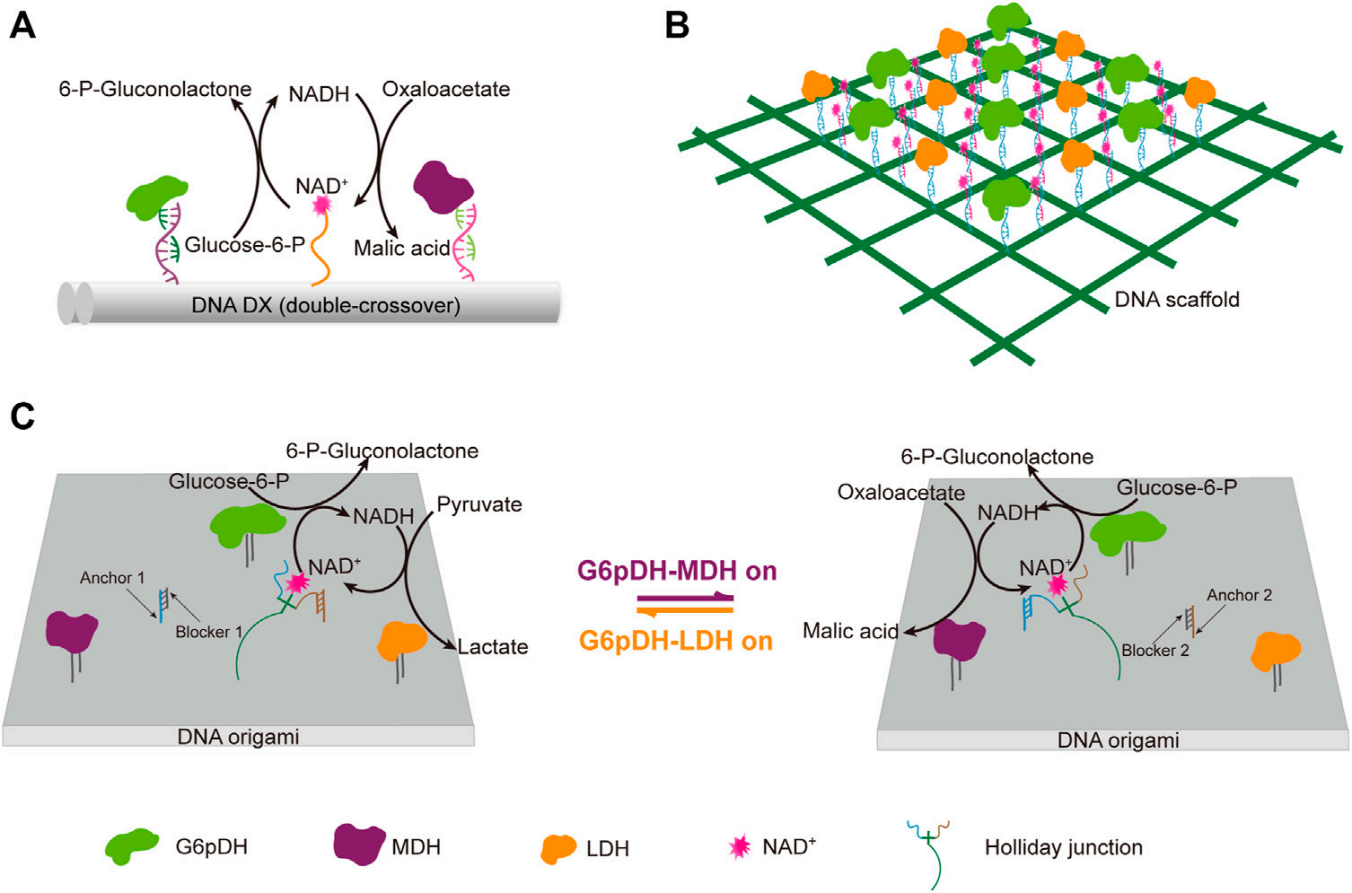
DNA architecture-guided assembly of multi-enzyme machineries with facilitated cofactor recycling. **(A)** Nanostructure complex with G6pDH, MDH, and NAD^+^ organized on a DNA double-crossover tile scaffold. **(B)** 2D enzyme network of G6pDH, LDH, and NAD^+^ organized on a 6 × 6 lattice DNA origami. **(C)** Enzyme pathway regulation system consisting of G6pDH, MDH, LDH, and a swing arm with a NAD^+^-coupled Holliday junction immobilized on a DNA origami platform. The addition of a blocker strand would release the Holliday junction from the corresponding anchor strand, and facilitate its binding with another anchor strand. In all status, the swinging arm served to facilitate the transport of redox intermediates NAD^+^/NADH between one of the enzyme pairs on the platform. G6pDH: Glucose-6-phosphate dehydrogenase, MDH: Malic dehydrogenase, LDH: Lactate dehydrogenase.

DNA scaffolds can be used for programmable immobilization of not only the enzymes, but also their cofactors. Yan group designed a swinging-arm nanostructure complex, in which a glucose-6-phosphate (G6PDH) and a malic dehydrogenase (MDH) were co-immobilized on a DNA double-crossover tile scaffold, and a NAD^+^-functionalized poly(T)_20_ oligonucleotide, was attached to the surface of the DNA tile in between these two enzymes, and behaved as a swinging-arm that facilitated hydride transfer between G6PDH and MDH (Figure 8A) (Fu et al., 2014). They demonstrated the substantial effect of the distance between the swinging arm and enzymes, and the relative orientation and stoichiometric ratio of NAD^+^ and enzymes on the catalytic performance of the system. They also found that the increased rigidity of the swinging arm caused by the hybridization with a complementary poly(A)_20_ oligonucleotide had an obvious negative impact on the catalytic activity. Later, they expanded this work to the construction of an artificial 2D enzyme network of G6PDH and lactate dehydrogenase (LDH), in which the enzymes were immobilized on the nodes of a wireframe DNA origami grid, and the cofactor NAD^+^-coupled duplex swinging arms were immobilized halfway between each pair of G6PDH and LDH (Figure 8B) (Yang et al., 2018). They found that the overall catalytic activity was highly dependent on the length of the swing arms, which transferred the hydride intermediate from one enzyme to another. They also compared the activity of G6PDH/LDH system immobilized on a 2D origami lattice with that of G6PDH/LDH system immobilized on a liner double-crossover DNA tile. When total enzyme concentrations were the same, the enzyme-origami complex showed higher activity due to the availability of more swinging NAD^+^ arms. This result was also in good agreement with their previous study with a similar complex, in which G6PDH/MDH system was immobilized on a 4 × 4 DNA tile scaffold (Fu et al., 2014). Later, they developed a system with both G6pDH/MDH and G6pDH/LDH enzyme pairs immobilized on a rectangular DNA origami scaffold, in which the cofactor NAD^+^ could controllably shift between these two enzyme pairs (Figure 8C) (Ke et al., 2016). A four-arm Holliday junction was made with one arm attached to a NAD^+^, one arm attached to the DNA origami platform, and the other two consisting of two single-stranded ends, which could selectively bind to the anchor DNAs immobilized in between those two enzyme pairs, so that the cofactor shift could be regulated through the binding of blocker DNAs with the anchor DNAs.

**FIGURE 9:**
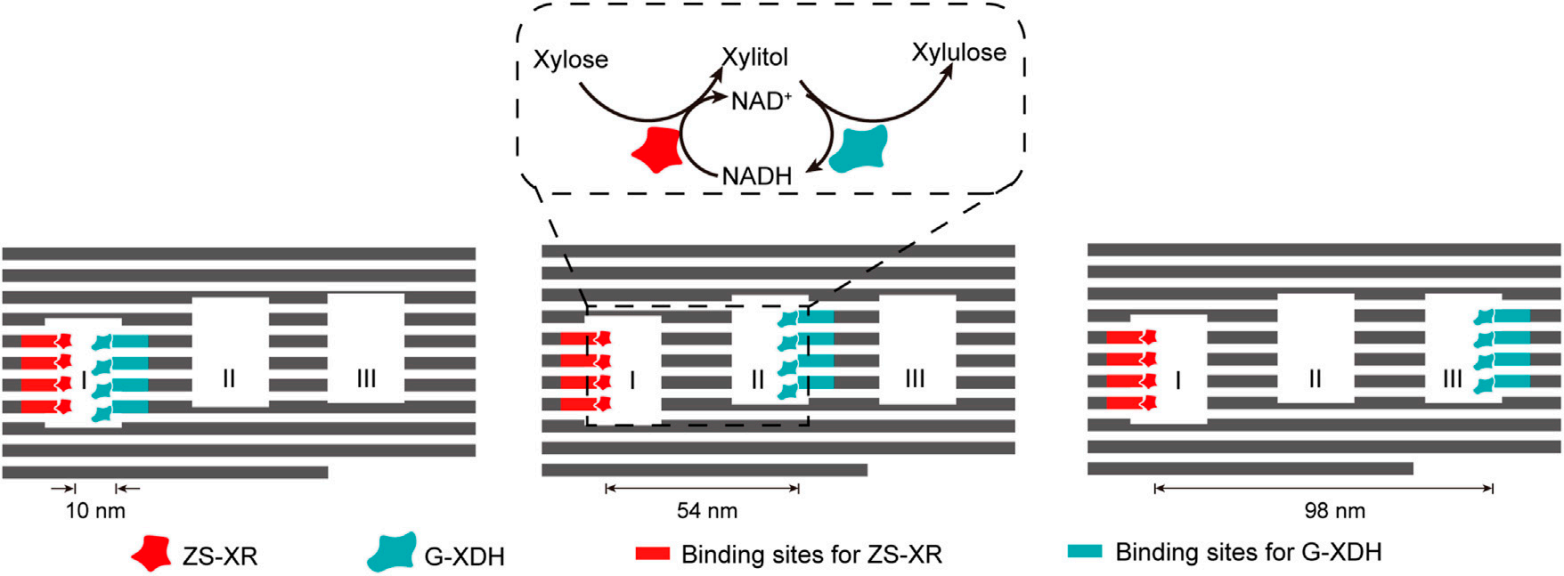
DNA-framed artificial enzyme cascade of the xylose metabolic pathway. DNA origami scaffold with three cavities were used to control the distance between the enzymes. Binding sites for ZS-XR and G-XDH were placed at varied spots on the inner surface of the three cavities. ZS-XR: Xylose reductase (XR) fused with a modular adaptor (ZS) consisting of a zif268 and a SNAP-tag, which can form a covalent linkage with benzylguanine modified DNA sequence; G-XDH: Xylitol dehydrogenase (XDH) fused with a basic leucine-zipper GCN4 protein. Zif268 and GCN4 are two DNA-binding proteins that specifically bind to different sequences.

The interaction between DNA binding proteins and their recognition sequences can also be well applied in the construction of multi-enzyme systems scaffolded by complex DNA nanostructures, simply by integrating and displaying these binding sequences on the surface of DNA nanostructures. By fusing the enzymes with zinc finger protein zif268 and basic leucine-zipper protein GCN4 respectively, Ngo et al. site-specifically assembled xylose reductase (XR) and xylitol dehydrogenase (XDH) of xylose metabolic pathway on DNA origami nanostructures with different distribution patterns of protein binding sites (Figure 9) (Ngo et al., 2016). While XR was anchored inside cavity I of the DNA scaffold, XDH was allocated to different cavities to modulate the inter-enzyme distance. The catalytic efficiency of the cascade reaction gradually increased when the inter-enzyme distance decreased from 98 nm to 10 nm.

There is barely any example of using RNA scaffolds for the *in vitro* construction of multi-enzyme systems, presumably due to the fact that RNA is not as stable and cheap as DNA. However, there are some examples of using RNA scaffolds for the *in vivo* construction of multi-enzyme systems, taking advantage of easy *in vivo* production of RNA *via* transcription and efficient co-transcriptional assembly of RNA nanostructures, which will be introduced in next section. Unnatural nucleic acid scaffolds have also not been used for the construction of multi-enzyme systems yet, since the efforts on the development of efficient tools to produce, amplify, and manipulate them, including unnatural nucleic acid polymerases, although already fruitful, are still in progress. The great potential of this field will be discussed in the end of this article.

## *In Vivo* Construction of Multi-Enzyme Systems With Nucleic Acid Frameworks for Efficient Cascade Biocatalysis

**FIGURE 10:**
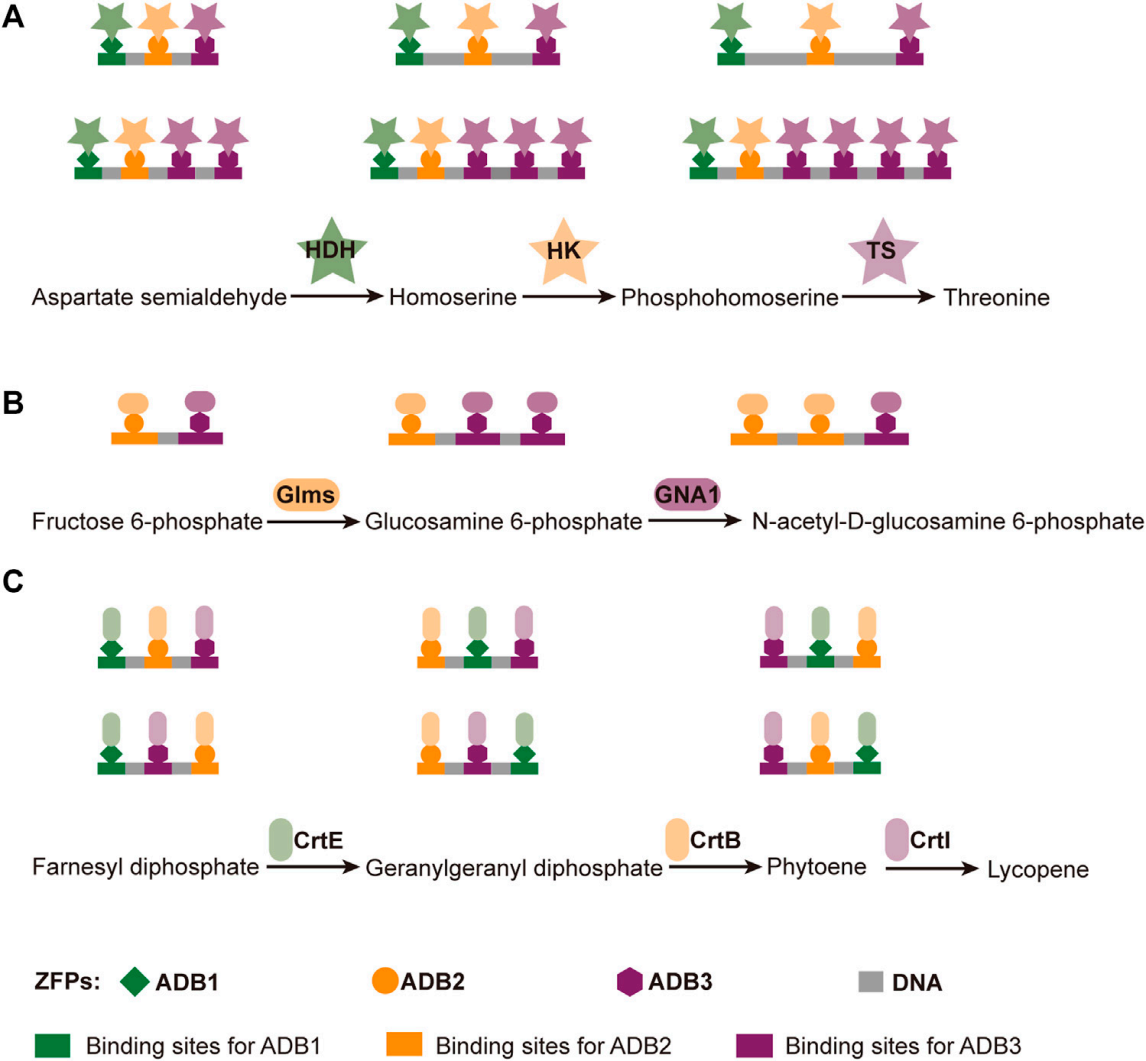
DNA scaffolded multi-enzyme systems for *in vivo* biosynthesis of different compounds: **(A)** L-threonine; **(B)** N-acetylglucosamine; **(C)** Lycopene. ZFPs: Zinc finger proteins; ADB: Artificial DNA binding domains; HDH: Homoserine dehydrogenase; HK: Homoserine kinase; TS: Threonine synthase; Glms: Glucosamine synthase; GNA1: *N*-acetylglucosamine *N*-acetyltransferase; CrtE: Geranylgeranyl diphosphate synthase; CrtB: Phytoene synthase; CrtI: Phytoene desaturase.

**FIGURE 11:**
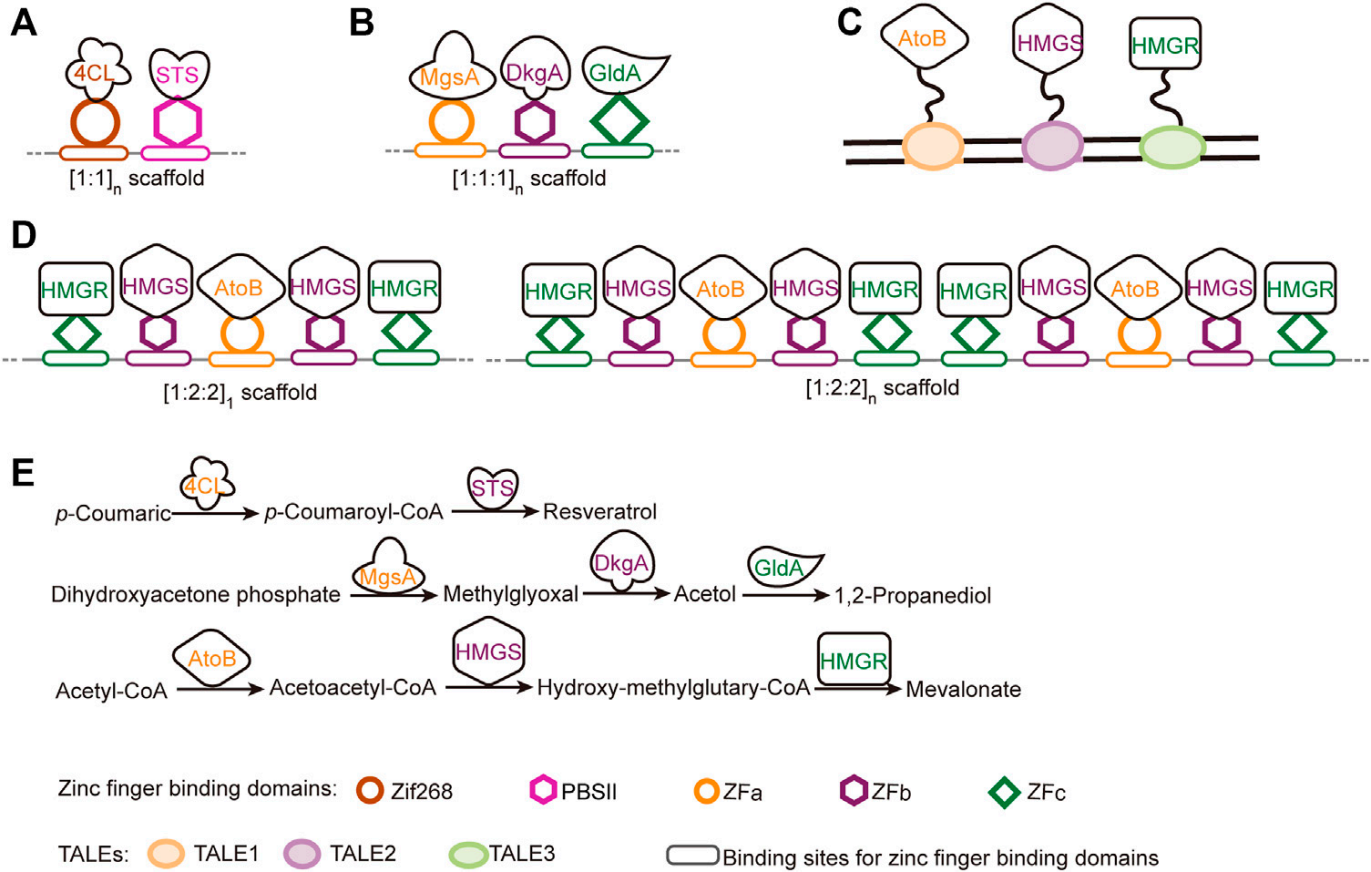
DNA-guided *in vivo* assembly of biosynthetic pathways with programmed enzyme arrangements. The relative positions and the stoichiometric ratio of the enzymes, and the repetition of the units were well controlled by arranging the binding sites for different DNA binding proteins. **(A)** (1:1)_n_ system developed for resveratrol biosynthesis. **(B)** (1:1:1)_n_ system developed for 1,2-propanediol (1,2-PD) biosynthesis. **(C)** Synthetic tri-enzymatic pathway for mevalonate production. **(D)** (1:2:2)_n_ system developed for mevalonate biosynthesis. The scaffolds were designed such that the first enzyme in the pathway was always flanked on both sides by the second and third enzymes, giving rise to a bidirectional pathway arrangement. **(E)** Biosynthesis pathways of resveratrol, 1,2-propanediol and mevalonate. Zif 268, PBSII, ZFa, ZFb, ZFc are five different zinc finger domains and their binding sequences are shown as rounded squares. 4CL: 4-Coumarate:CoA ligase; STS: Stilbene synthase; MgsA: Methylglyoxal synthase; DkgA: 2,5-Diketo-D-gluconic acid reductase; GldA: Glycerol dehydrogenase; AtoB: Acetoacetyl-CoA thiolase; HMGS: Hydroxy-methylglutaryl-CoA synthase; HMGR: Hydroxy-methylglutaryl-CoA reductase.

Whole cell biocatalysts have been broadly used in bioindustry for the production of valuable chemicals, and their catalytic efficiency can be significantly improved *via in vivo* construction of multi-enzyme systems with key enzymes in the metabolic pathways (Dueber et al., 2009; Conrado et al., 2012; Qu et al., 2019). Biomacromolecules that can be synthesized in living cells and genetically programmed for predefined structures and assemblies are obviously the best options of scaffold materials for the construction of intracellular multi-enzyme systems. Besides the *in vitro* applications, DNA scaffolds are also extensively used as templates to guide the *in vivo* assembly of multi-enzyme systems, in which the enzymes are genetically fused to DNA binding proteins with varied recognition sequences. Zinc finger proteins, which are among the most abundant proteins in eukaryotic organism and involved in various cellular activities, consist of repeated zinc binding motifs containing cysteine and histidine ligands (Laity et al., 2001). Many of zinc finger proteins recognize and bind to dsDNAs with specific sequences to function as transcription factors, which provides perfect tools for programmable organization of proteins on dsDNA scaffolds. Lee et al. constructed and optimized a zinc finger protein/DNA scaffold-based multi-enzyme system for the production of L-threonine (Figure 10A) (Lee et al., 2013). Plasmid containing specific binding sequences of zinc finger proteins and plasmid harboring the genes of zinc finger protein-fused enzymes, which are involved in L-threonine biosynthesis, were constructed and co-transformed into *Escherichia coli* (*E. coli*) cells. After being expressed in the cells, the zinc finger protein-fused enzymes spontaneously assembled onto the scaffold plasmid. They then optimized stoichiometric ratios of the enzymes and intervals between the enzymes for higher L-threonine production. With the optimal system, the production time of L-threonine was reduced by over 50%, and the growth rate of the host cells was also enhanced, presumably due to reduced accumulation of toxic intermediates. Chen and coworkers reported the application of similar strategy on spatial modulation of pathway enzymes for improved production of N-acetylglucosamine in *Bacillus subtilis* (Figure 10B) (Liu et al., 2014). The zinc finger protein-directed intracellular assembly of multi-enzyme systems also proved effective for modulating various other metabolic pathways and improving the production of many other compounds, including lycopene, resveratrol, 1,2-propanediol and mevalonate. (Figure 10C and Figure 11) (Conrado et al., 2012; Xu X. et al., 2020). TALEs are transcription factors which contain highly conserved tandem repeats of 34-aa sequences that are essential for sequence-specific DNA recognition and binding, and have been engineered and broadly applied in targeted genome editing and regulation of gene expression (Sanjana et al., 2012). Taking advantages of the precise binding between TALE proteins and DNA sequences, a DNA-guided multi-enzyme system can be assembled *in vitro* or *in vivo* by simply fusing enzymes with TALE proteins and immobilizing them onto a DNA scaffold with multiple TALE binding sequences. Zhu et al. reported the *in vivo* construction of TALE-based multi-enzyme system for the synthesis of indole-3-acetic acid (Zhu et al., 2016). In this system, tryptophan-2-mono-oxygenase (IAAM) and indole-3-acetimide hydrolase (IAAH) were genetically fused with rationally designed TALEs, and site-specifically immobilized on a plasmid DNA scaffold containing TALE recognition sequences with different intervals. Later, they reported the construction of a synthetic tri-enzymatic pathway in *E. coli* (Xie et al., 2019). After optimization of the scaffold and the plasmid copy number, this synthetic tri-enzyme pathway demonstrated significantly increased productivity of mevalonate from acetyl CoA.

Up to now, not many examples of applying complex nucleic acid nanostructures for *in vivo* construction of multi-enzyme systems have been reported yet, mainly due to the lack of efficient and user-friendly methods for *in vivo* production and assembly of singles-stranded nucleic acid components, as well as methods for *in vivo* immobilization of enzymes of interest site-specifically onto these nanostructures. Sliver et al. pioneered an effort to construct multi-enzyme systems *in vivo* based on RNA nanostructures (Delebecque et al., 2011). RNAs with programmed sequences were transcribed and assembled into discrete, 1D, and 2D nanostructures in living cells, and the enzymes fused with protein adaptors were immobilized on specific sites through the binding of these adaptors with their aptamers, which were prefabricated into those RNA nanostructures. By organizing the proteins in hydrogen synthetic pathway with this strategy, they successfully optimized the production of hydrogen. Later, with similar strategy, they demonstrated the application of various RNA binding domain (RBD)-aptamer sets in localizing enzymes on *in vivo* constructed 2D RNA scaffolds for enhanced pentadecane and succinate production (Sachdeva et al., 2014).

## Summary and Perspectives

In the past decades, a variety of elegant artificial structures based on nucleic acids have been designed and constructed, and some of them have found broad application in the construction of elegant nanomaterials or as highly programmable scaffolds for multi-enzyme systems. In this article, we started from the introduction of basic characteristics and properties of natural and unnatural nucleic acids, which demonstrated the uniqueness and superiority of nucleic acids to be used as structural materials. The progress in the development of polymerases for the efficient synthesis of nucleic acids with unnatural moieties, which are essential for broader application of unnatural nucleic acids, was also discussed. We then summarized representative approaches in the application of DNA, RNA, and unnatural nucleic acids for the construction of various artificial nanostructures, ranging from simple one or two-dimensional structures to complicated 3D origami nanostructure assemblies. In the end, we reviewed the construction of multi-enzyme systems using nucleic acid structures as scaffolds, as well as the application of these multi-enzyme systems for efficient catalysis of cascade reactions, both *in vitro* and *in vivo*.

Rapid development of DNA and RNA nanotechnology has enabled the construction and broad application of various nucleic acid nanostructures. However, more efforts still need to be made to make the procedures and the computational programs for the design and construction of complicated nanostructures more robust and user-friendly (Andronescu et al., 2003; Jabbari et al., 2015; Jun et al., 2019). For broader practical and even industrial applications of the nucleic acid nanostructures, the techniques for massive and cost-effective production of DNA and RNA building blocks also need to be developed and optimized (Chandler et al., 2020; Geary et al., 2021). Recently, biomass DNA directly extracted from living organisms was successfully converted into biodegradable materials, ranging from gels to plastics, at large scales with very low costs (Wang et al., 2020; Han et al., 2021). While these approaches demonstrated the feasibility of acquiring and applying massive amount of DNA raw materials from nature for the construction of functional materials, development of novel technologies for efficient synthesis of longer and more accurate oligonucleotides, such as enzymatic *de novo* synthesis technology, will facilitate the acquirement of more and better oligonucleotide sequences to be used as components for the construction of nucleic acid nanostructures in the future (Jensen and Davis, 2018; Perkel, 2019; Eisenstein, 2020).

In order to immobilize and organize two or more enzymes for efficient catalysis of cascade reactions, multi-enzyme systems have employed various materials to fabricate the scaffolds, including inorganic materials, organic frameworks, and biological materials. Among those, nucleic acid has proven one of the most promising scaffold materials. Numerous artificial structures have been constructed with nucleic acids, and many of them are already well suited for the construction of multi-enzyme systems for efficient catalysis of cascade reactions *in vitro* or *in vivo*. However, these systems are still far from perfect, and a lot of efforts need to be made to further improve their performance. For instance, new chemistry may be developed to achieve efficient site-specific immobilization of the enzymes onto the scaffolds and precise control of the orientation of the enzymes, which is obviously very important for the accessibility of substrates or intermediates to the active sites. Smarter nucleic acid nanostructures may also be designed for better regulation of the stoichiometric ratio and relative positions of the enzymes based on their kinetic parameters to achieve optimal overall catalytic performance. Development of better computational tools to assist the design, assembly, and behavior simulation of versatile nucleic acid nanostructures will also greatly promote these efforts (de Llano et al., 2020; Poppleton et al., 2020). Despite being constructed with very high complexity and applied for guiding the assembly of highly programmable multi-enzyme systems *in vitro*, complex DNA origami nanostructures are rarely used as scaffolds for *in vivo* construction of multi-enzyme systems. Most of the nucleic acid scaffold-based multi-enzyme system constructed *in vivo* are still simply based on the immobilization of DNA-binding protein elements on linear double strands of DNA. Further exploitation of methods for constructing complicated nucleic acid nanostructures *in vivo* and combining these nanostructures with well-designed nucleic acid-binding proteins may lead to much better scaffolds for *in vivo* assembly of highly programmable multi-enzyme systems, and facilitate the engineering of cellular metabolic pathways.

Although natural nucleic acid frameworks have already been broadly used in the construction of nanomaterials or as the scaffolds of multi-enzyme systems, there still remain many challenges in the construction and application of better nucleic acid scaffolds that fulfill the requirements of more programmable and robust nanostructures or multi-enzyme systems. For example, the number of nucleobases in natural DNA and RNA is quite limited compared to the number of amino acids in protein, which results in limited sequences, structures, and designability of DNA and RNA. Moreover, natural nucleic acids have low tolerance against nuclease degradation and reduced stability under various chemical or physical conditions, which restricts the application of nucleic acid nanostructures or multi-enzyme systems with nucleic acid scaffolds in biological solutions or harsh environments. The fast development of unnatural components has provided a generous toolbox for the structural and functional expansion and augmentation of nucleic acids, and thus offered a great potential for the design and construction of better nucleic acid-based nanostructures and multi-enzyme scaffolds. As mentioned above, newly developed unnatural base pairs are already able to pair, replicate and transcribe as efficiently as natural base pairs (Betz et al., 2017; Feldman et al., 2019; Ouaray et al., 2020). The introduction of these unnatural base pairs into nucleic acids will undoubtedly increase the possible sequences, structures, properties, and functionalities of nucleic acids, and thus provide more options for components of nanostructures and scaffolds of multi-enzyme systems. The site-specifically incorporated unnatural base pairs may also be chemically modified with various functional groups, side chains, or linkers, and thus provide much more controllable sites or handles for the immobilization, organization, and regulation of biomacromolecules, including enzymes. The introduction of unnatural sugar-phosphate backbones will not only expand the possible structures and functionalities of nucleic acids, but can also significantly increase the stability of the nucleic acid scaffolds under different chemical, physical, or biological conditions, and thus lead to broader application scenarios for the nanostructures and multi-enzyme systems. While chemical synthesis and polymerase engineering underlies the development and *in vitro* application of unnatural nucleic acids, the efforts on construction of semi-synthetic organisms have opened the gate for *in vivo* application of unnatural nucleic acids (Zhang et al., 2017a; Zhang et al., 2017b; Fischer et al., 2020; Hashimoto et al., 2021; Manandhar et al., 2021), so we can confidently expect the broad application of unnatural components in future construction of nucleic acid scaffolds for building nanostructures and multi-enzyme machineries, both *in vitro* and *in vivo*.
